# Supplementary material for: A dish-like molecular architecture for dynamic ultralong room-temperature phosphorescence through reversible guest accommodation
Source: Nat Commun. 2022 Dec 2;13:7423. doi: 10.1038/s41467-022-35155-y (PMC9715674; doi:10.1038/s41467-022-35155-y)
Supplement: Supplementary file 1 — Supplementary Information [file 41467_2022_35155_MOESM1_ESM.pdf]

Supplementary Information

A Dish-Like Molecular Architecture for Dynamic Ultralong Room-Temperature Phosphorescence through Reversible Guest Accommodation

Li et al.

Supplementary Methods

Theoretical calculations

The theoretical calculations based on CP molecules were performed by using the time-dependent density functional theory (TD-DFT) method at the B3LYP/6-31G* level in the Gaussian 16 program (version A.03) based on single crystal structures.[1] The molecular geometry optimization of linear alkyl bromides was performed at the B3LYP/6-31G* level. The natural transition orbital (NTO) analysis was extracted based on TD-DFT results with the aid of Multiwfn package.[2] Noncovalent interactions (NCI) of intermolecular interactions analyses based on single-crystal structures were carried out by using Multiwfn software with independent gradient model (IGM) and plotted via VMD software (version 1.9.3).[3] The spin-orbit coupling (SOC) between singlets and triplets were evaluated by using PySOC program based on the TD-DFT results.[4]

Synthesis

10-(4-((4-fluorophenyl)sulfonyl)phenyl)-10*H*-phenothiazine (FP)

The synthesis and purification of compound FP were similar to our previous paper.[5] Sodium hydride (0.84 g, 35.0 mmol) was added into a solution of 10*H*-phenothiazine (2.11 g, 10.6 mmol) in DMF (30 mL). After the mixture was stirred under nitrogen atmosphere for 15 min, bis(p-fluorophenyl)sulfone (3.00 g, 11.8 mmol) was added. Then the mixture was heated up to 70°C and stirred for 6 h. After the mixture was cooled down to room temperature, the crude product was purified by silica gel column chromatography with DCM/*n*-hexane (v/v = 1:3) as eluent. Compound FP was obtained as a yellow solid in 65% yield (3.0 g). ^{1}H NMR (400 MHz, $CDCl_3$): δ 7.97–7.85 (m, 2H), 7.77–7.71 (m, 2H), 7.47–7.41 (m, 2H), 7.35–7.29 (m, 4H), 7.21 (ddd, J = 7.8, 6.0, 2.7 Hz, 2H), 7.18–7.12 (m, 2H), 7.11–7.06 (m, 2H); ^{13}C NMR (101 MHz, $CDCl_3$): δ 165.82, 163.28, 149.01, 140.21, 137.89, 132.82, 132.16, 129.52, 129.42, 128.72, 128.30, 126.77, 125.70, 125.59, 115.93, 115.71, 115.36; HRMS (m/z): $[M]^+$ calcd. for $C_{24}H_{16}FNO_2S_2$, 433.0606; found, 433.0606.

10-(4-((4-(9*H*-carbazol-9-yl)phenyl)sulfonyl)phenyl)-10*H*-phenothiazine (CP)

Sodium hydride (0.28 g, 11.7 mmol) was added into a solution of carbazole (0.58 g, 3.5 mmol) in DMF (30 mL). After the mixture was stirred under nitrogen atmosphere for 15 min, FP (1.00 g, 2.3 mmol) was added. Then the mixture was heated up to 70°C and stirred for 6 h. After the mixture was cooled down to room temperature, the crude product was purified by silica gel column chromatography with DCM/*n*-hexane (v/v = 1:1) as eluent. Compound CP was obtained as a white solid in 75% yield (1.0 g). ^{1}H NMR (500 MHz, DMSO-d_6): δ 8.24 (d, J = 7.7 Hz, 2H), 8.15 (d, J = 8.6 Hz, 2H), 7.98–7.80 (m, 4H), 7.56 (d, J = 7.6 Hz, 2H), 7.49 (d, J = 8.2 Hz, 2H), 7.46–7.38 (m, 6H), 7.30 (dt, J = 9.1, 4.7 Hz, 4H), 7.13 (d, J = 9.1 Hz, 2H); ^{13}C NMR (126 MHz, DMSO-d_6): δ 149.70, 141.67, 140.84, 140.45, 139.83, 132.89, 132.79, 130.13, 129.56, 129.34, 128.44, 127.62, 127.13, 127.01, 126.90, 123.71, 121.33, 121.12, 116.49, 110.35; HRMS (m/z): $[M]^+$ calcd. for $C_{36}H_{24}N_2O_2S_2$, 580.1279; found, 580.1278.

10,10'-(sulfonylbis(4,1-phenylene))bis(10*H*-phenothiazine) (2P)

The synthesis and purification of compound 2P were similar to our previous paper.[6] Sodium hydride (0.14 g, 5.8 mmol) was added into a solution of phenothiazine (1.16 g, 5.8 mmol) in DMF (30 mL). After stirred for 15 min under a nitrogen atmosphere, bis(p-fluorophenyl)sulfone (0.50 g, 2.0 mmol)

was added. Then the mixture was heated up to 110°C and stirred for 12 h. After the mixture was cooled down to room temperature, the crude product was purified by silica gel column chromatography with DCM/*n*-hexane (v/v = 1:4) as eluent. Compound 2P was obtained as a pale-yellow solid in 75% yield (0.9 g). ^{1}H NMR (400 MHz, $CDCl_3$): δ 7.81–7.71 (m, 4H), 7.43–7.35 (m, 4H), 7.31–7.23 (m, 4H), 7.21–7.14 (m, 8H), 7.14–7.07 (m, 4H); ^{13}C NMR (101 MHz, $CDCl_3$): δ 148.21, 140.59, 133.99, 131.43, 128.60, 128.05, 126.70, 125.26, 124.54, 116.97; HRMS (m/z): $[M]^+$ calcd. for $C_{36}H_{24}N_2O_2S_3$, 612.1000; found, 612.1006.

9,9'-(sulfonylbis(4,1-phenylene))bis(9*H*-carbazole) (2C)

Sodium hydride (0.14 g, 5.8 mmol) was added into a solution of carbazole (0.96 g, 5.8 mmol) in DMF (30 mL). After stirred for 15 min under a nitrogen atmosphere, bis(p-fluorophenyl)sulfone (0.50 g, 2.0 mmol) was added. Then the mixture was heated up to 110°C and stirred for 12 h. After the mixture was cooled down to room temperature, the crude product was purified by silica gel column chromatography with DCM/*n*-hexane (v/v = 1:3) as eluent. Compound 2C was obtained as a white solid in 65% yield (0.7 g). ^{1}H NMR (500 MHz, DMSO-d_6): δ 8.34 (d, J = 8.6 Hz, 4H), 8.25 (d, J = 7.7 Hz, 4H), 7.99 (d, J = 8.6 Hz, 4H), 7.54 (d, J = 8.3 Hz, 4H), 7.44 (t, J = 7.7 Hz, 4H), 7.32 (t, J = 7.3 Hz, 4H); ^{13}C NMR (126 MHz, DMSO-d_6): δ 142.27, 139.79, 139.33, 130.24, 127.77, 127.04, 123.79, 121.42, 121.15, 110.40; HRMS (m/z): $[M]^+$ calcd. for $C_{36}H_{24}N_2O_2S$, 548.1558; found, 548.1561.

10-(4-((4-(1*H*-benzo[*f*]indol-1-yl)phenyl)sulfonyl)phenyl)-10*H*-phenothiazine (CP-ISO)

Sodium hydride (0.05 g, 2.0 mmol) was added into a solution of 1*H*-benzo[*f*]indole (0.10 g, 0.6 mmol) in DMF (10 mL). After the mixture was stirred under nitrogen atmosphere for 15 min, FP (0.17 g, 0.4 mmol) was added. Then the mixture was heated up to 70°C and stirred for 6 h. After the mixture was cooled down to room temperature, the crude product was purified by silica gel column chromatography with DCM/*n*-hexane (v/v = 1:1) as eluent. Compound CP-ISO was obtained as a white solid in 88% yield (0.2 g). ^{1}H NMR (400 MHz, $CDCl_3$): δ 8.16 (s, 1H), 8.12–8.06 (m, 2H), 8.05 (s, 1H), 7.95 (dd, J = 6.0, 3.4 Hz, 1H), 7.87 (dd, J = 6.1, 3.3 Hz, 1H), 7.85–7.79 (m, 2H), 7.74–7.67 (m, 2H), 7.51 (d, J = 3.5 Hz, 1H), 7.44 (d, J = 7.6Hz, 2H), 7.42–7.37 (m, 2H), 7.35–7.30 (m, 4H), 7.21 (ddd, J = 7.8, 5.5, 3.2 Hz, 2H), 7.16–7.10 (m, 2H), 6.85 (d, J = 3.1 Hz, 1H); ^{13}C NMR (101 MHz, $CDCl_3$): δ 149.05, 143.24, 140.23, 138.42, 134.93, 132.88, 132.26, 130.25, 130.18, 130.03, 128.84, 128.71, 128.66, 128.33, 127.49, 127.06, 126.81, 125.73, 125.68, 123.89, 122.97, 122.83, 118.50, 115.33, 105.60, 104.58; HRMS m/z: $[M]^+$ calculated for $C_{36}H_{24}N_2O_2S_2$, 580.1279; found: 580.1278.

2-nitro-1,1'-biphenyl

The synthesis and purification of compound 2-nitro-1,1'-biphenyl were similar to the previous paper.[7] A 2.0 M aqueous K_2CO_3 solution (12.0 mL) was added to a THF solution (30 mL) containing 1-bromo-2-nitrobenzene (2.00 g, 10.0 mmol) and phenylboronic acid (1.67 g, 15.0 mmol) and stirred for 15 mins under a nitrogen atmosphere. $Pd(PPh_3)_4$ catalyst (0.02 g) was added and stirred at 80°C for 6 h. After the mixture was cooled down to room temperature, the crude product was purified by silica gel column chromatography with DCM/*n*-hexane (v/v=1:10) as eluent. Compound 2-nitro-1,1'-biphenyl was obtained as a white solid in 76% yield (1.5 g). ^{1}H NMR (400 MHz, $CDCl_3$): δ 7.88 (dd, J = 8.0, 0.9 Hz, 1H), 7.61 (td, J = 7.6, 1.3 Hz, 1H), 7.51–7.44 (m, 5H), 7.42–7.36 (m,

2H); ^{13}C NMR (101 MHz, $CDCl_3$): δ 148.75, 136.93, 135.63, 131.92, 131.44, 128.22, 127.78, 127.73, 127.40, 123.53; HRMS m/z: $[M]^+$ calculated for $C_{12}H_9NO_2$, 199.0633; found: 199.0629.

9*H*-carbazole (Cz-Lab)

The synthesis and purification of compound Cz-Lab were similar to the previous paper.[7] triphenylphosphine (3.94 g, 120.0 mmol) was added to a *o*-DCB solution (20 mL) containing 2-nitro-1,1'-biphenyl (1.00 g, 40.0 mmol) and stirred for 15 mins under a nitrogen atmosphere. Then, the mixture was stirred at 180°C for 24 h. After the mixture was cooled down to room temperature, the crude product was purified by silica gel column chromatography with DCM/*n*-hexane (v/v=1:5) as eluent. Compound Cz-Lab was obtained as a white solid in 60% yield (0.5 g). ^{1}H NMR (400 MHz, DMSO-d_6): δ 11.24 (s, 1H), 8.10 (d, *J* = 7.8 Hz, 2H), 7.47 (d, *J* = 8.1 Hz, 2H), 7.42–7.32 (m, 2H), 7.19–7.09 (m, 2H); ^{13}C NMR (101 MHz, DMSO-d_6): δ 139.50, 125.32, 122.18, 119.97, 118.29, 110.73; HRMS m/z: $[M]^+$ calculated for $C_{12}H_9N$, 167.0735; found: 167.0730.

10-(4-((4-(9*H*-carbazol-9-yl)phenyl)sulfonyl)phenyl)-10*H*-phenothiazine (CP-Lab)

Sodium hydride (0.14 g, 5.9 mmol) was added into a solution of Cz-Lab (0.29 g, 1.8 mmol) in DMF (20 mL). After the mixture was stirred under nitrogen atmosphere for 15 min, FP (0.50 g, 1.2 mmol) was added. Then the mixture was heated up to 70°C and stirred for 6 h. After the mixture was cooled down to room temperature, the crude product was purified by silica gel column chromatography with DCM/*n*-hexane (v/v = 1:1) as eluent. Compound CP-Lab was obtained as a white solid in 60% yield (0.8 g). ^{1}H NMR (500 MHz, DMSO-d_6): δ 8.25 (d, *J* = 7.7 Hz, 2H), 8.16 (d, *J* = 8.6 Hz, 2H), 7.91 (dd, *J* = 8.8, 3.0 Hz, 4H), 7.57 (d, *J* = 7.6Hz, 2H), 7.49 (t, *J* = 8.0 Hz, 2H), 7.46–7.38 (m, 6H), 7.32 (t, *J* = 7.1 Hz, 4H), 7.13 (d, *J* = 9.0 Hz, 2H); ^{13}C NMR (126 MHz, DMSO-d_6): δ 149.05, 141.02, 140.17, 139.80, 139.17, 132.21, 132.18, 129.50, 128.97, 128.71, 127.80, 126.97, 126.49, 126.37, 126.29, 123.06, 120.69, 120.48, 115.79, 109.71; HRMS m/z: $[M]^+$ calculated for $C_{36}H_{24}N_2O_2S_2$, 580.1279; found: 580.1280.

Supplementary Figures

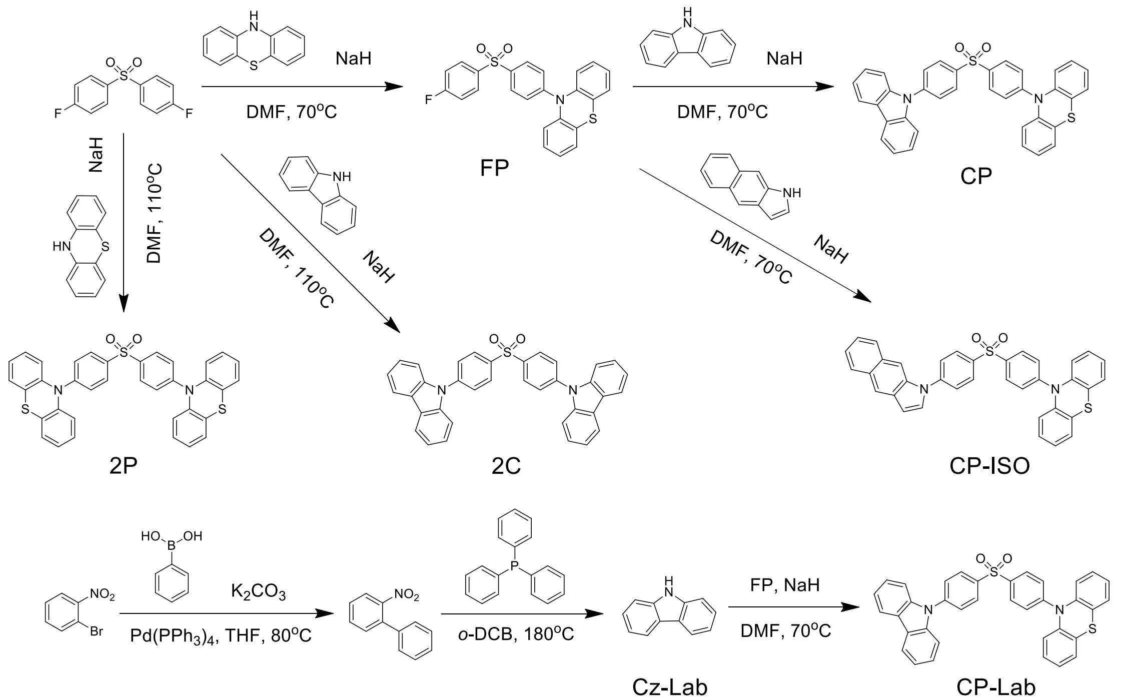

Supplementary Figure 1 | Synthetic routes of target molecules.

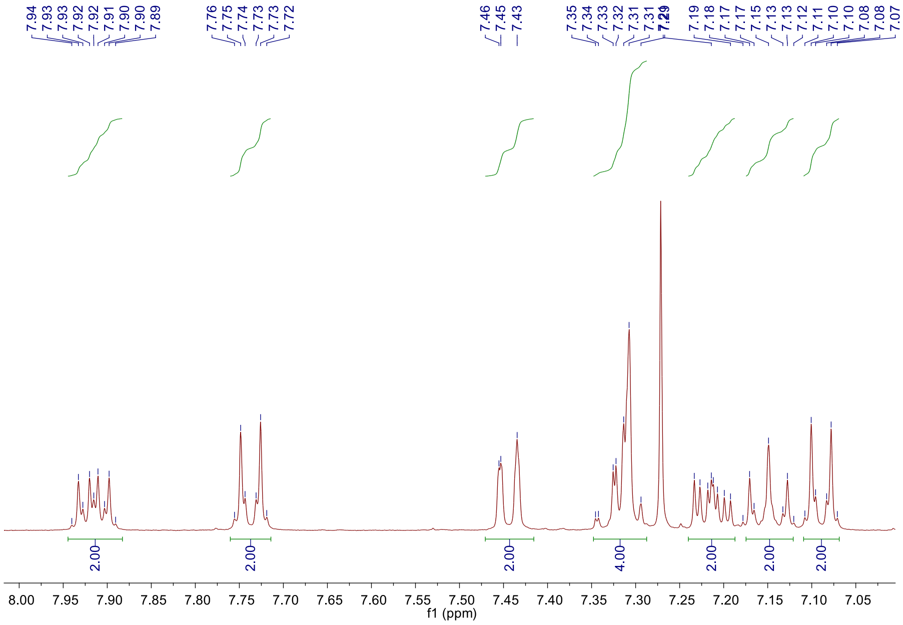

Supplementary Figure 2 | ^{1}H NMR spectrum of FP in $CDCl_3$.

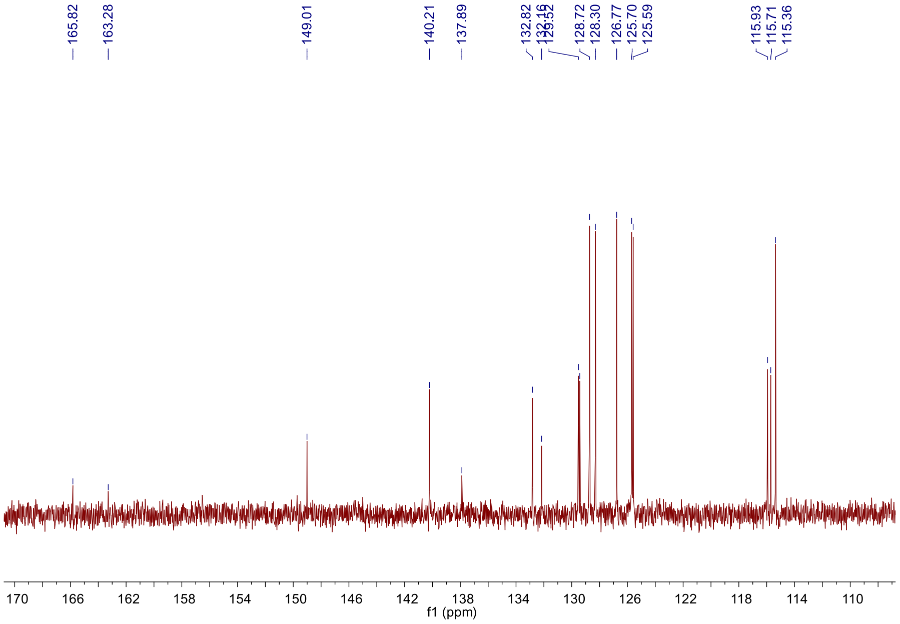

Supplementary Figure 3 | ^{13}C NMR spectrum of FP in $CDCl_3$.

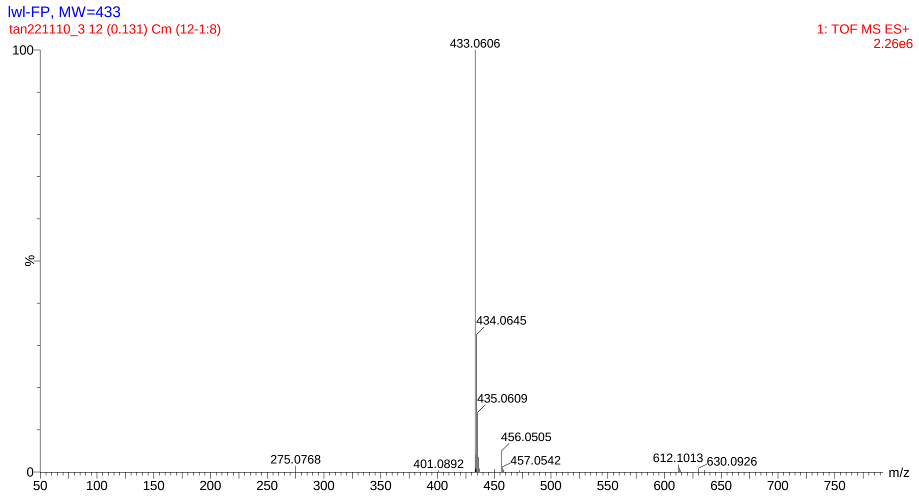

Supplementary Figure 4 | HRMS of the FP molecule.

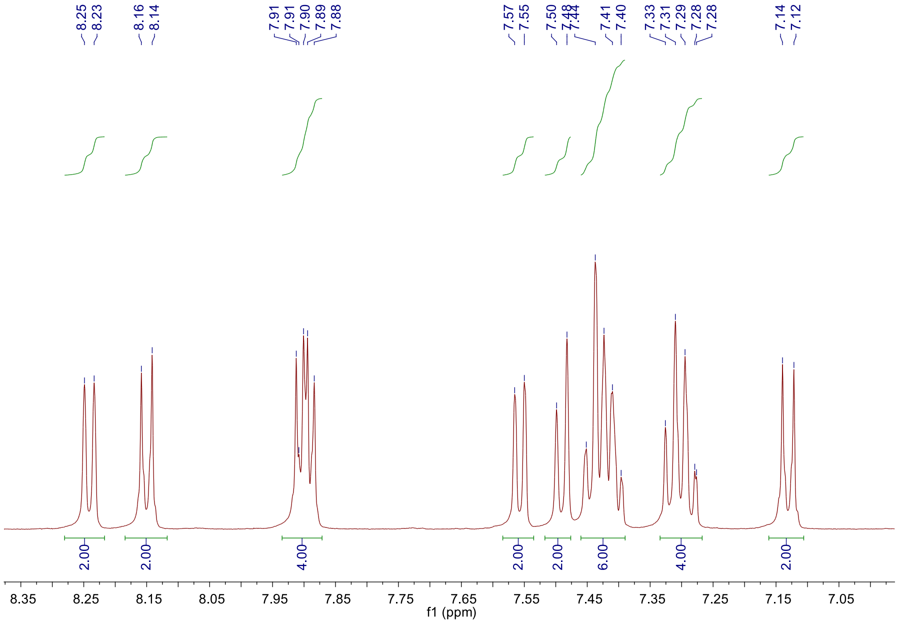

Supplementary Figure 5 | ^{1}H NMR spectrum of CP in DMSO-d_6.

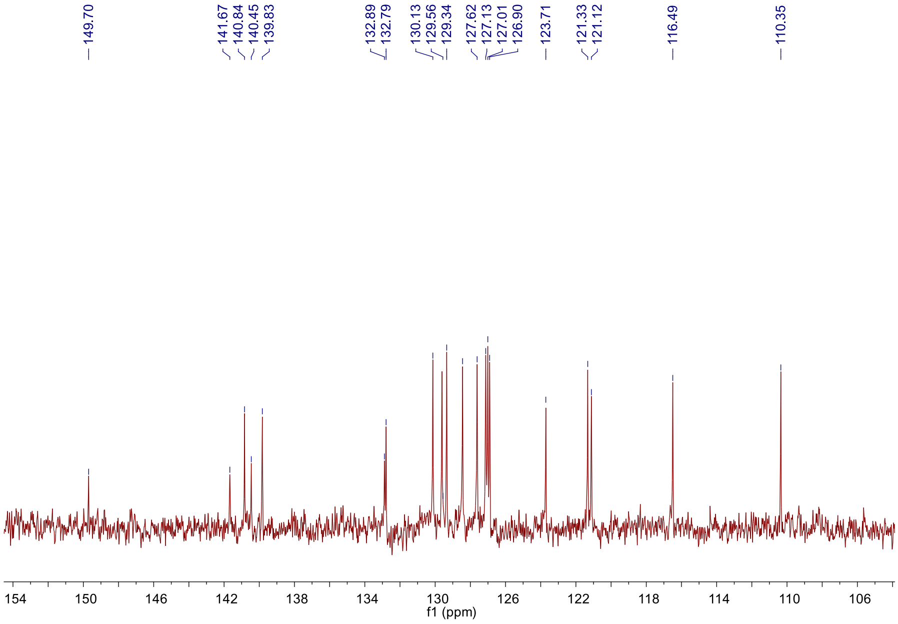

Supplementary Figure 6 | ^{13}C NMR spectrum of CP in DMSO-d_6.

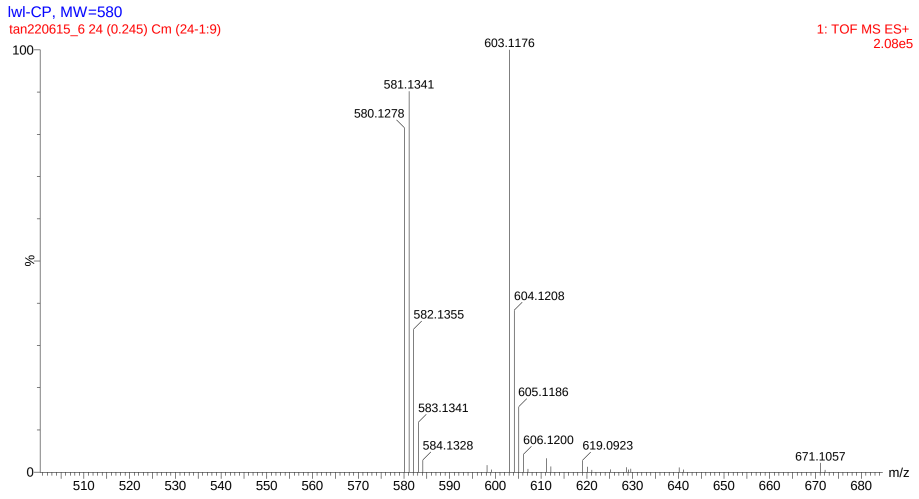

Supplementary Figure 7 | HRMS of the CP molecule.

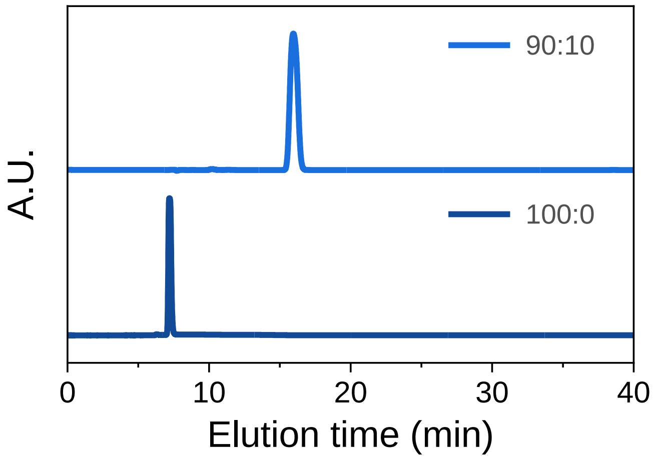

Supplementary Figure 8 | HPLC spectra of the CP molecule with acetonitrile-water as eluent in ratios of 100/0 and 90/10 (v/v).

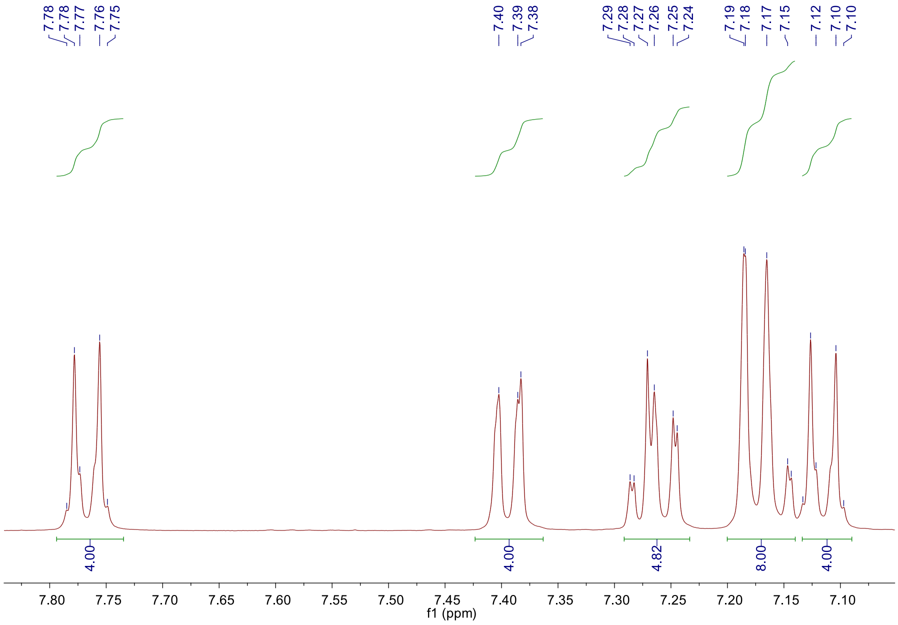

Supplementary Figure 9 | ^{1}H NMR spectrum of 2P in $CDCl_3$.

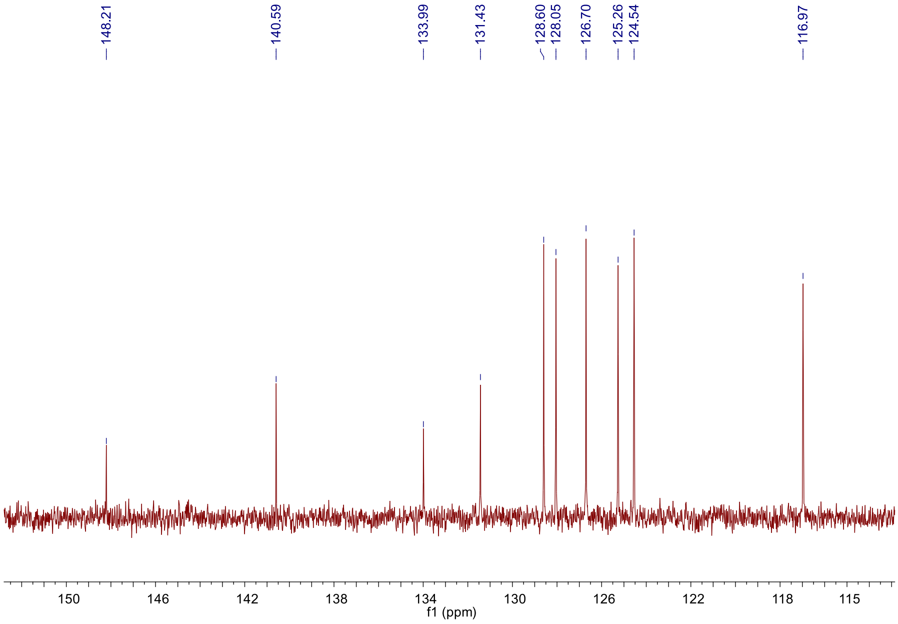

Supplementary Figure 10 | ^{13}C NMR spectrum of 2P in $CDCl_3$.

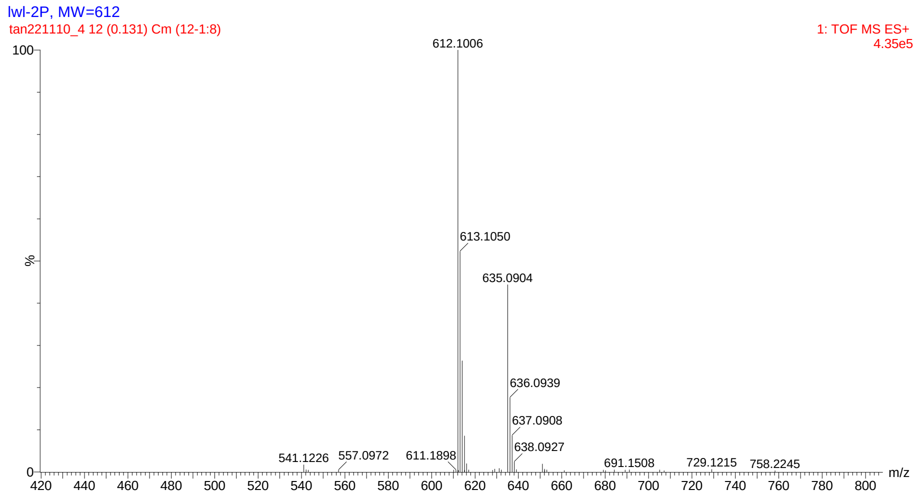

Supplementary Figure 11 | HRMS of the 2P molecule.

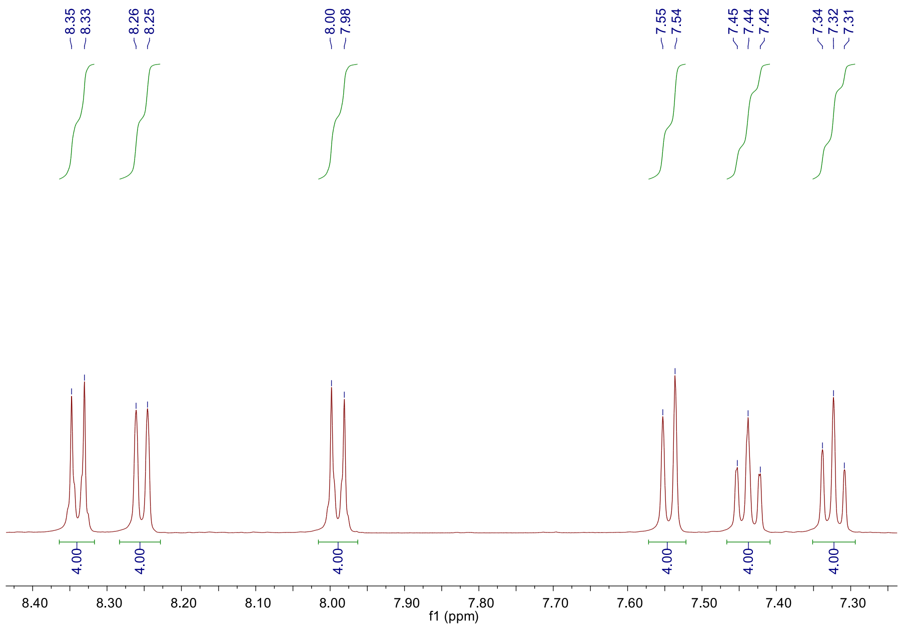

Supplementary Figure 12 | ^{1}H NMR spectrum of 2C in DMSO-d_6.

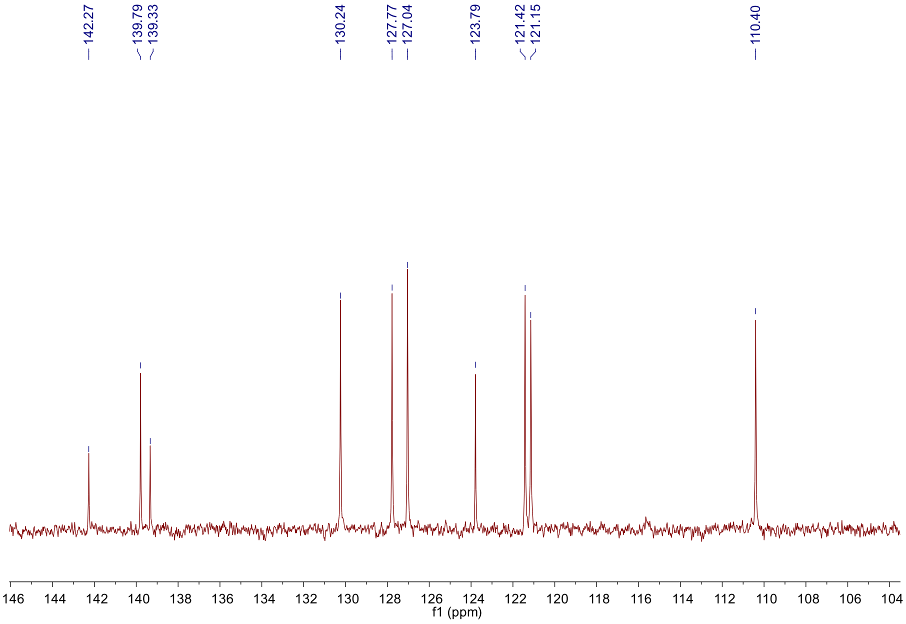

Supplementary Figure 13 | ^{13}C NMR spectrum of 2C in DMSO-d_6.

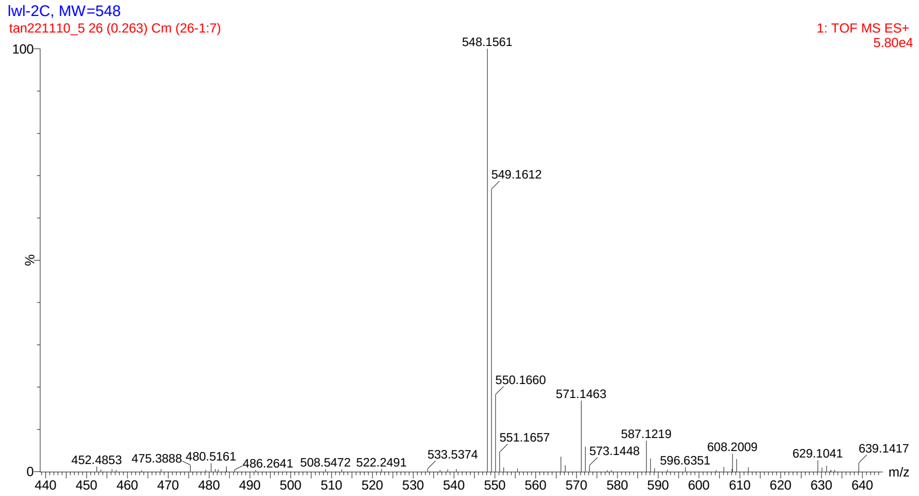

Supplementary Figure 14 | HRMS of the 2C molecule.

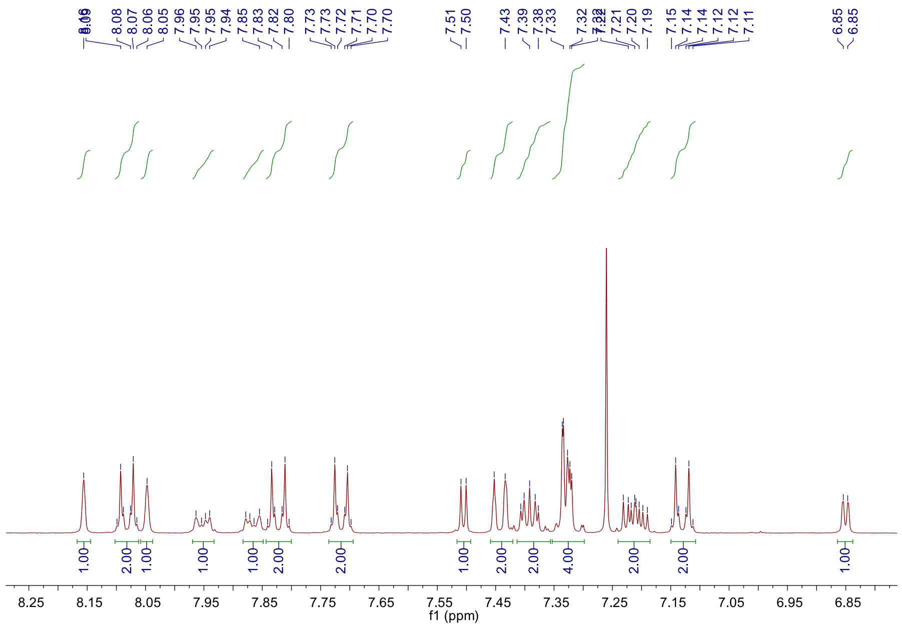

Supplementary Figure 15 | ^{1}H NMR spectrum of CP-ISO in $CDCl_3$.

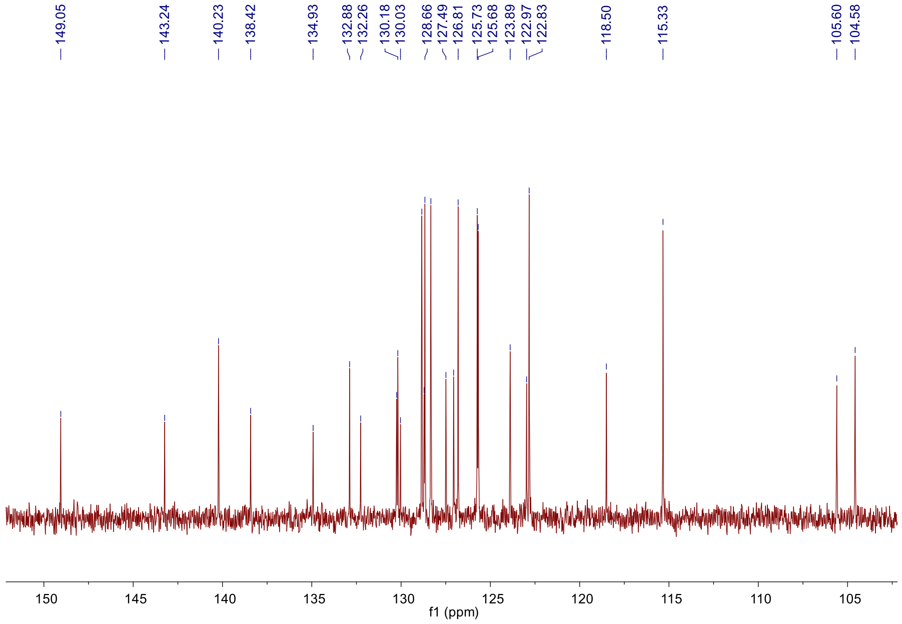

Supplementary Figure 16 | ^{13}C NMR spectrum of CP-ISO in $CDCl_3$.

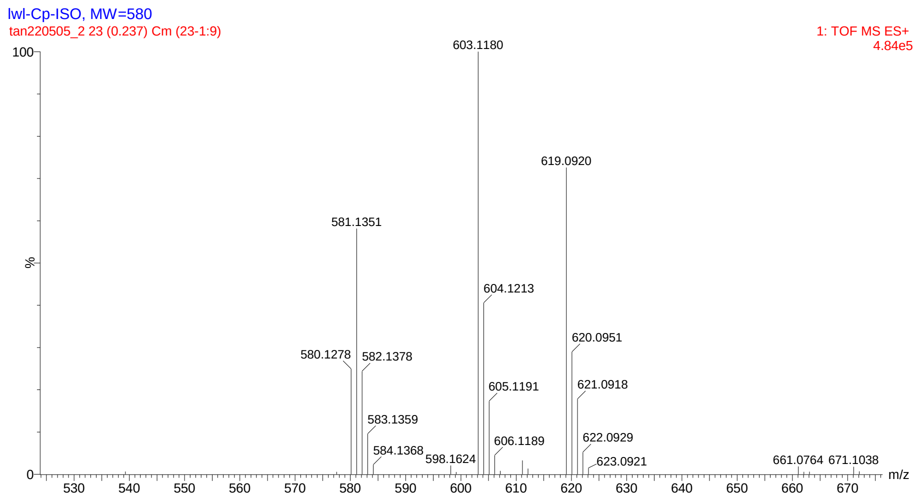

Supplementary Figure 17 | HRMS of the CP-ISO molecule.

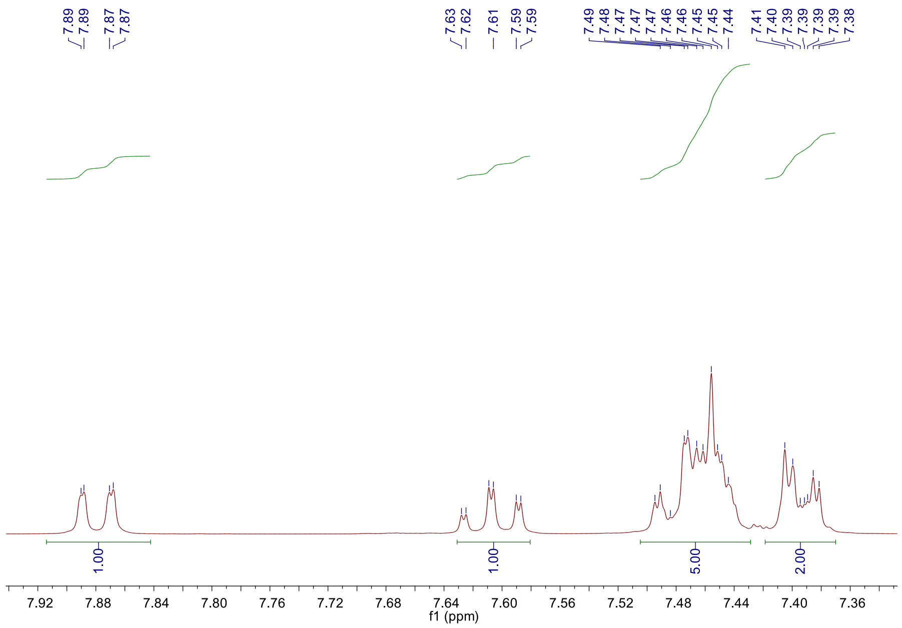

Supplementary Figure 18 | ^1H NMR spectrum of 2-nitro-1,1'-biphenyl in $CDCl_3$.

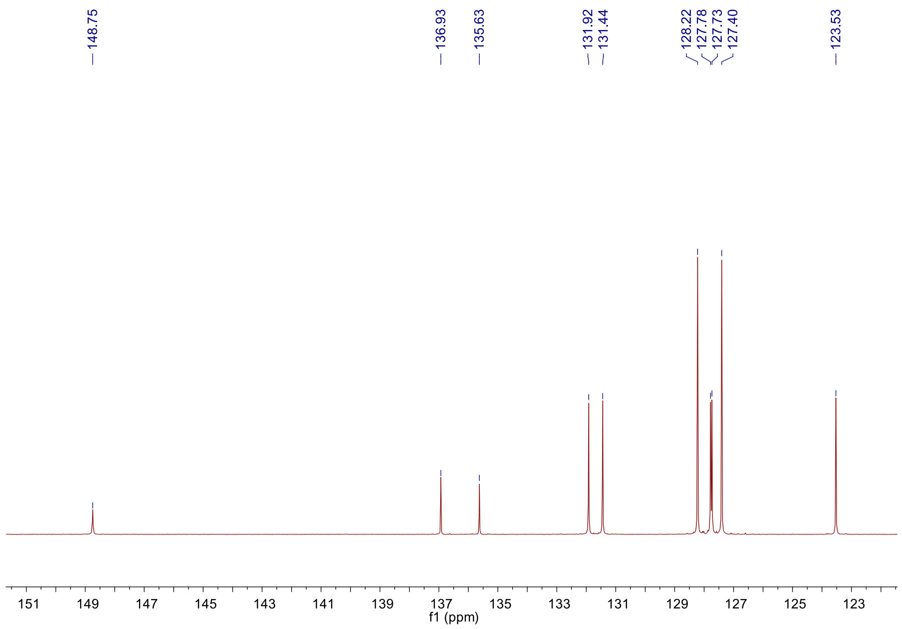

Supplementary Figure 19 | ^{13}C NMR spectrum of 2-nitro-1,1'-biphenyl in $CDCl_3$.

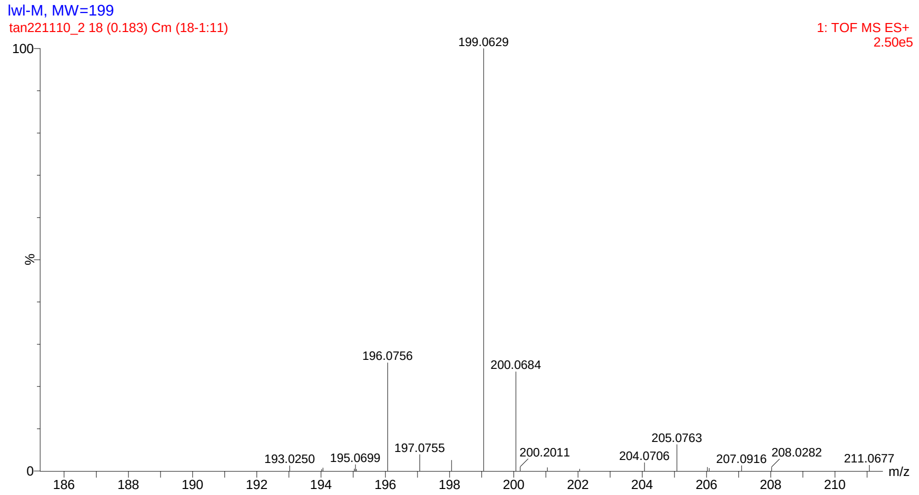

Supplementary Figure 20 | HRMS of the 2-nitro-1,1'-biphenyl molecule.

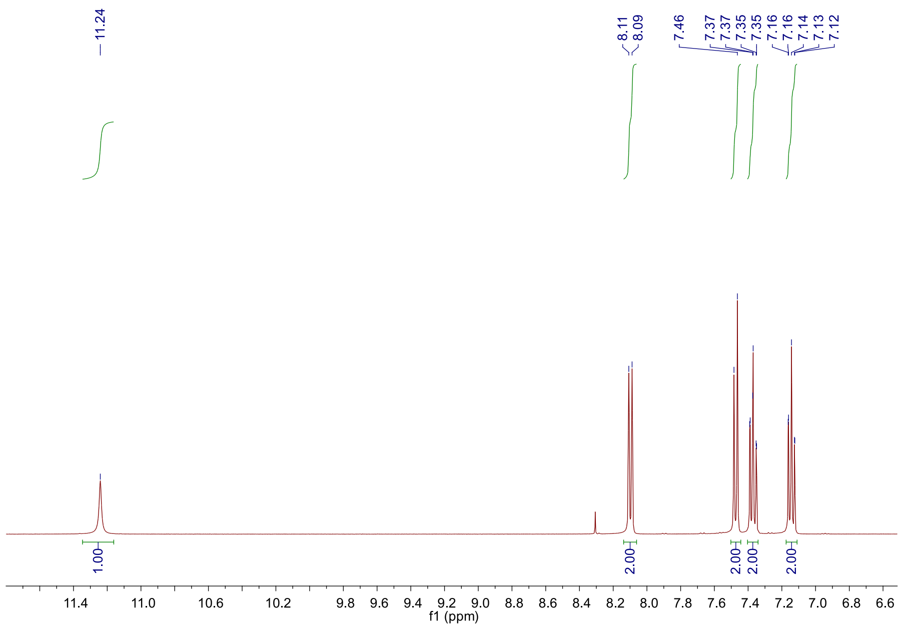

Supplementary Figure 21 | ^{1}H NMR spectrum of Cz-Lab in DMSO-d_6.

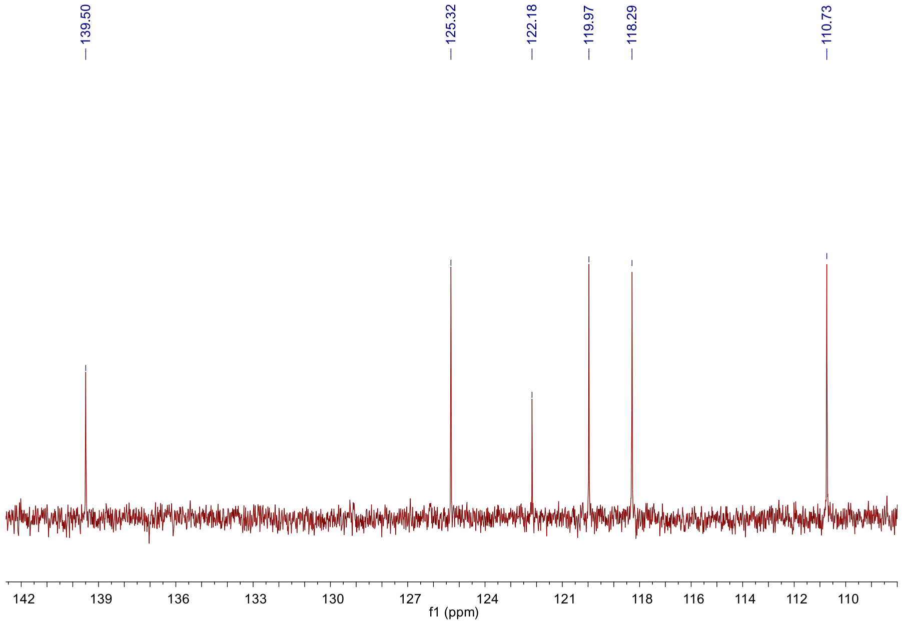

Supplementary Figure 22 | ^{13}C NMR spectrum of Cz-Lab in DMSO-d_6.

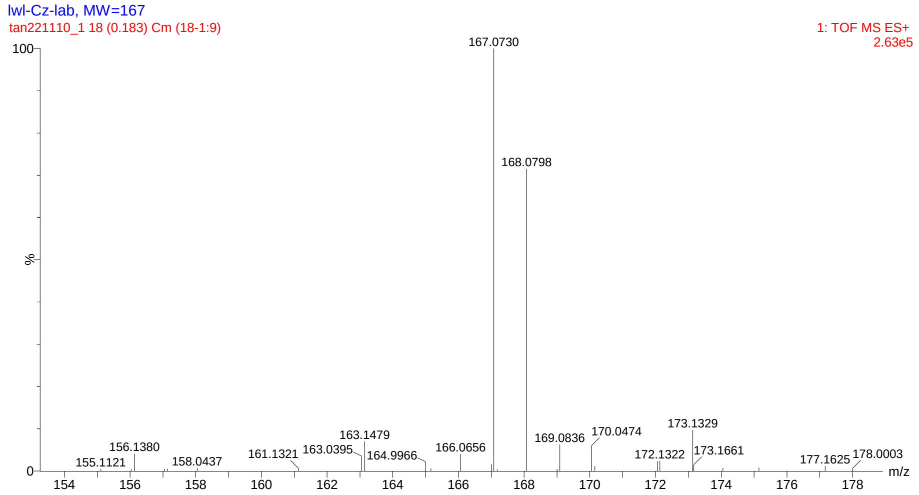

Supplementary Figure 23 | HRMS of the Cz-Lab molecule.

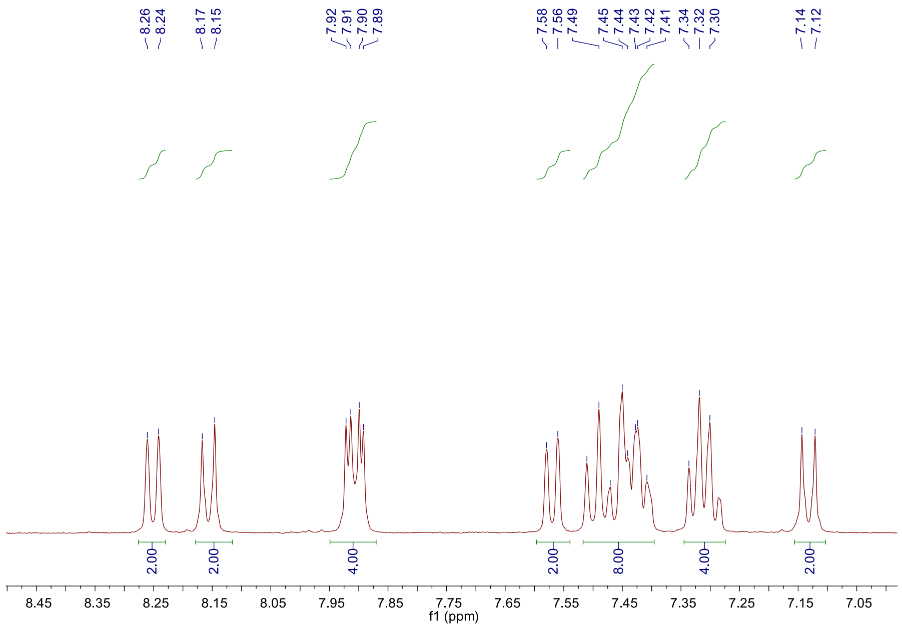

Supplementary Figure 24 | ^{1}H NMR spectrum of CP-Lab in DMSO-d_6.

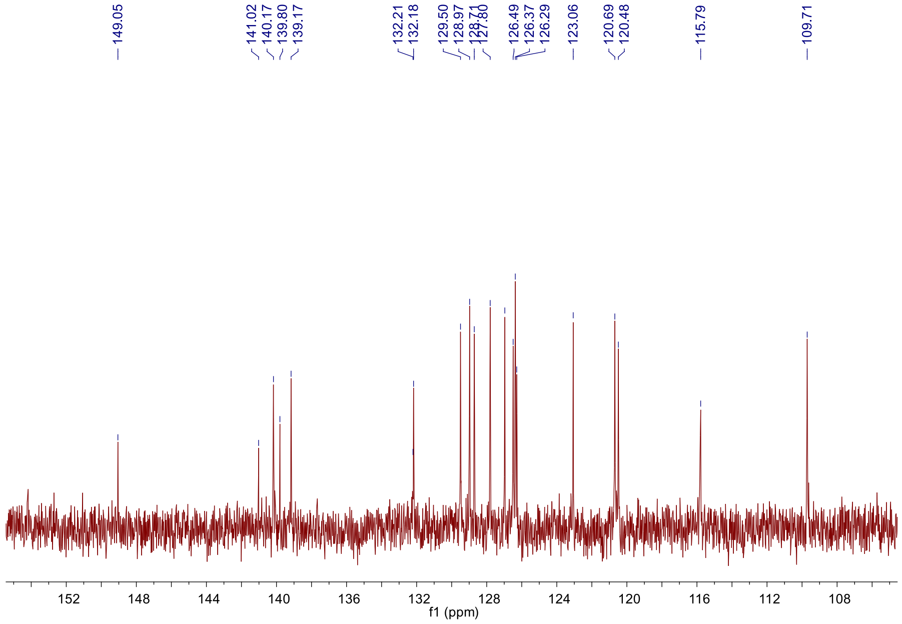

Supplementary Figure 25 | ^{13}C NMR spectrum of CP-Lab in DMSO-d_6.

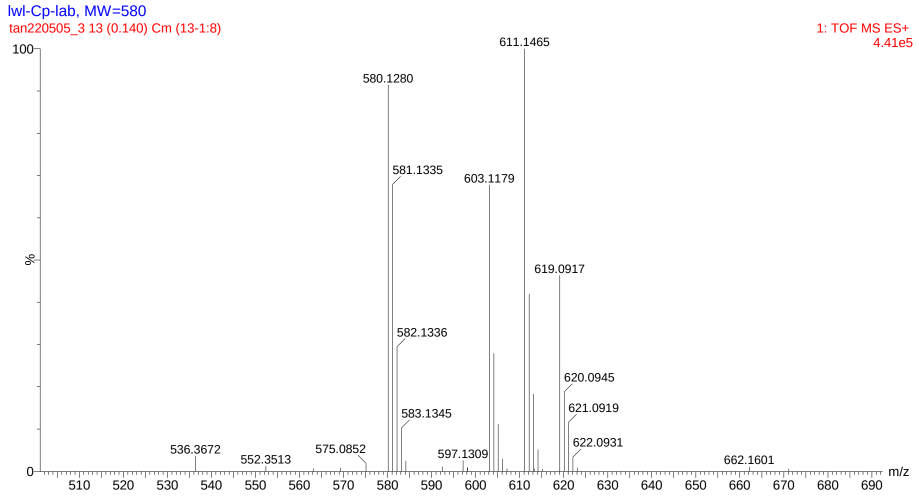

Supplementary Figure 26 | HRMS of the CP-Lab molecule.

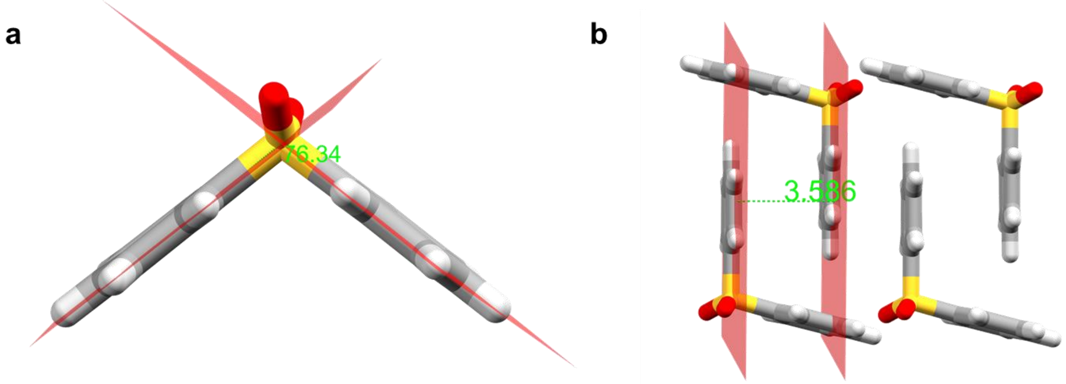

Supplementary Figure 27 | The **a,** dihedral angle and **b,** intermolecular π–π interaction in the single-crystal structure of diphenyl sulfone.[8]

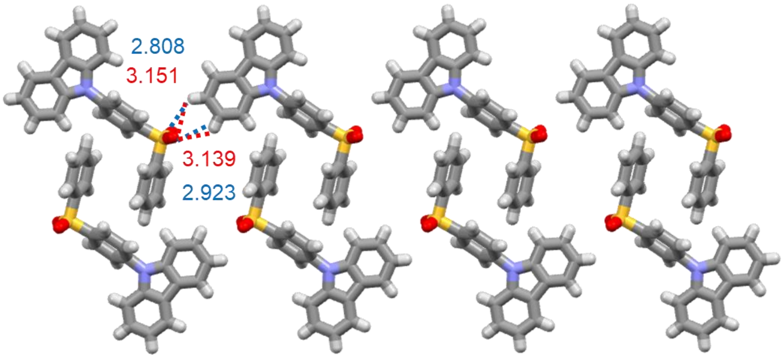

Supplementary Figure 28 | The single-crystal structure of CH.[9]

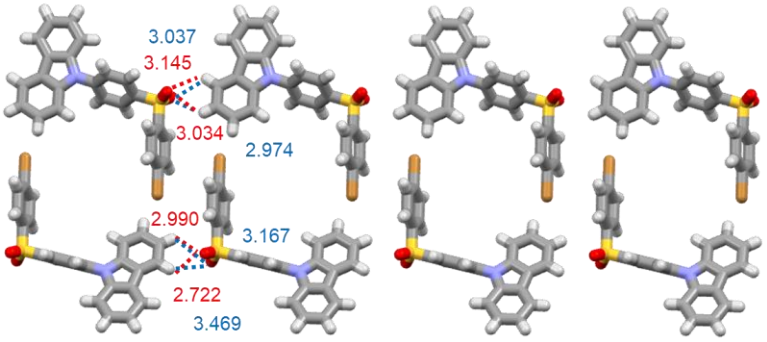

Supplementary Figure 29 | The single-crystal structure of CBr.[9]

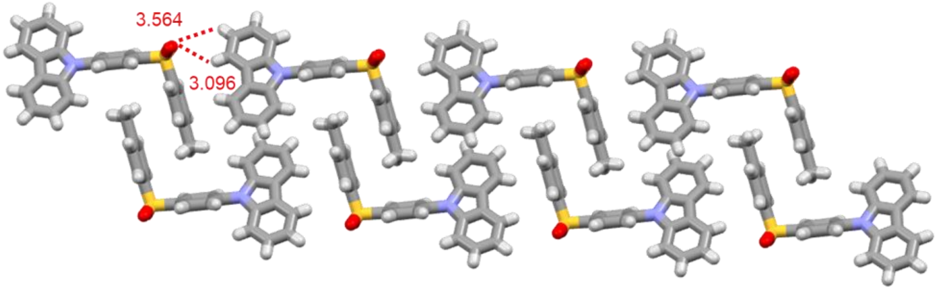

Supplementary Figure 30 | The single-crystal structure of CM.[10]

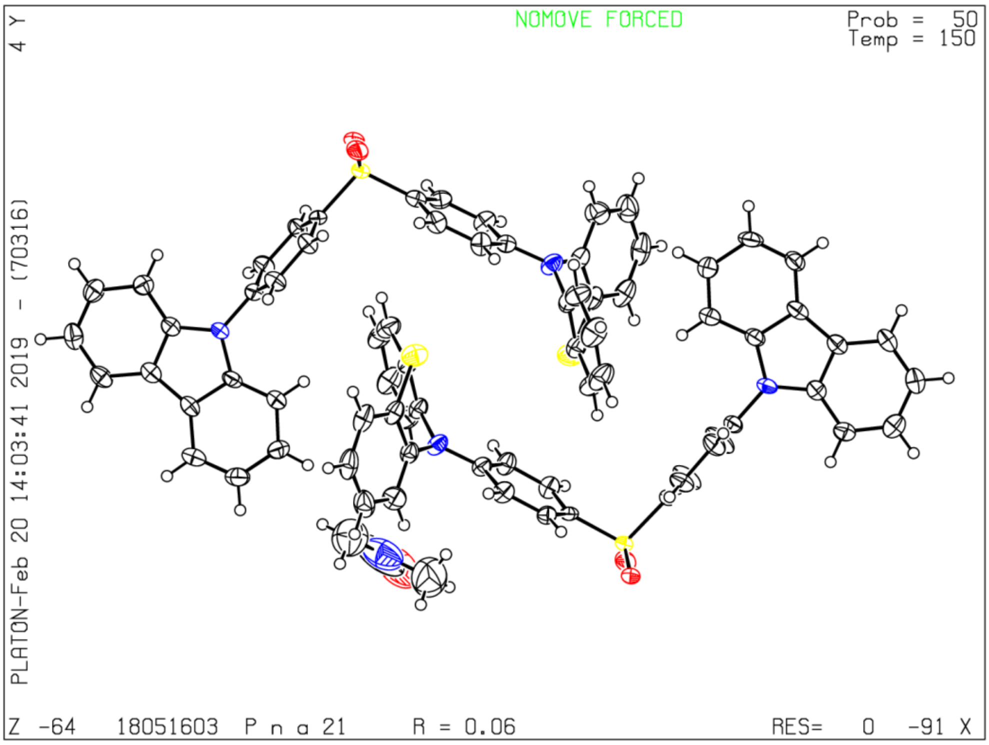

Supplementary Figure 31 | The single-crystal structure figure with probability ellipsoid of CP-DMF at the 50% level. Color code: white, C; red, O. White circles represent H atoms.

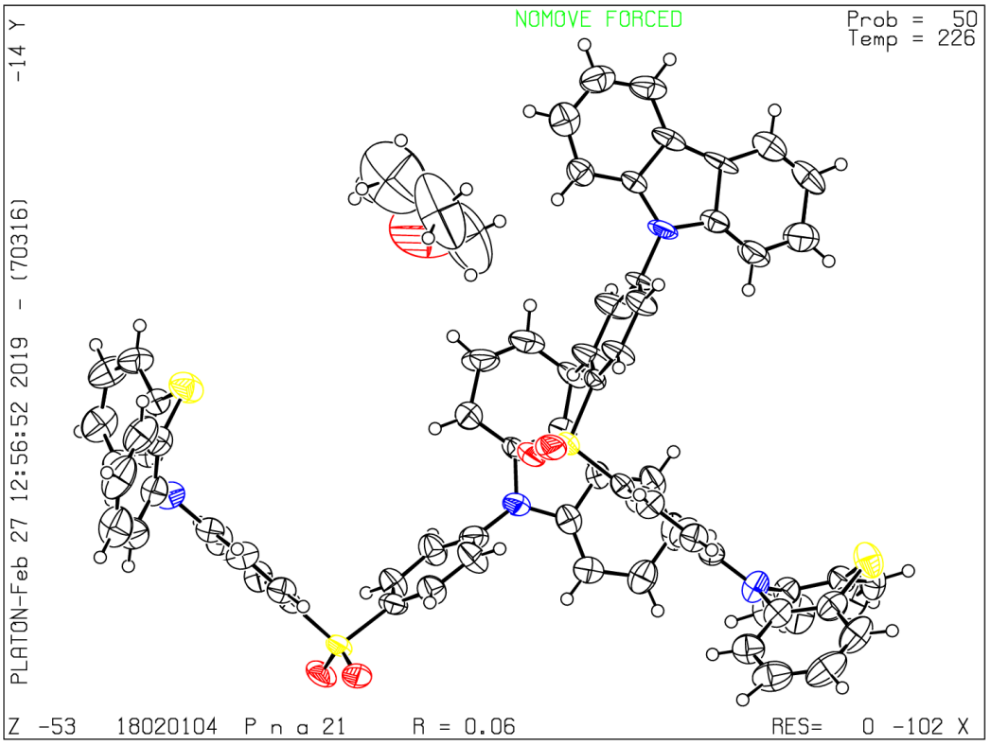

Supplementary Figure 32 | The single-crystal structure figure with probability ellipsoid of CP-THF at the 50% level. Color code: white, C; red, O. White circles represent H atoms.

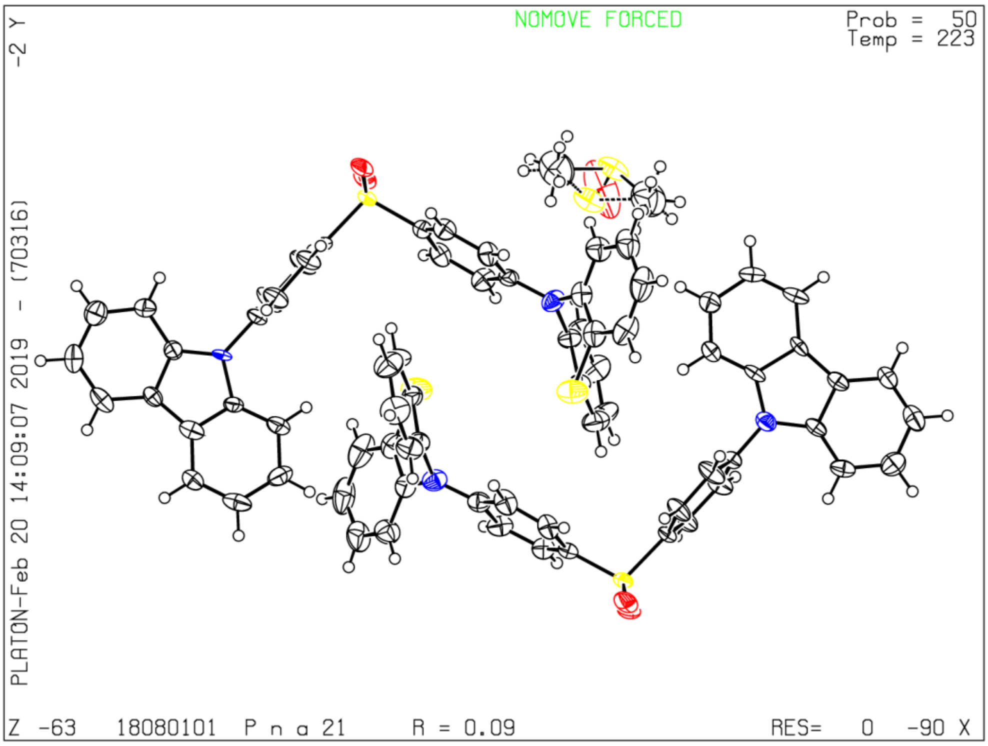

Supplementary Figure 33 | The single-crystal structure figure with probability ellipsoid of CP-DMSO at the 50% level. Color code: white, C; red, O. White circles represent H atoms.

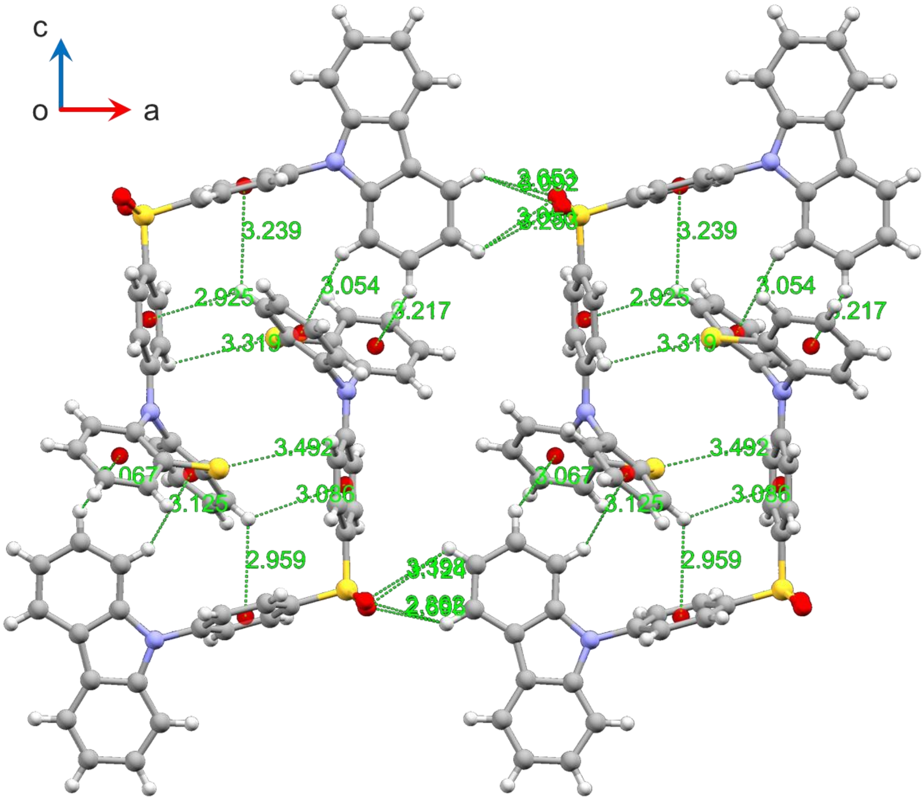

Supplementary Figure 34 | The intermolecular noncovalent interactions of the dish-like molecular architecture in the single-crystal structure of CP-DMF. Color code: grey, C; red, O; blue, N; yellow, S; white, H.

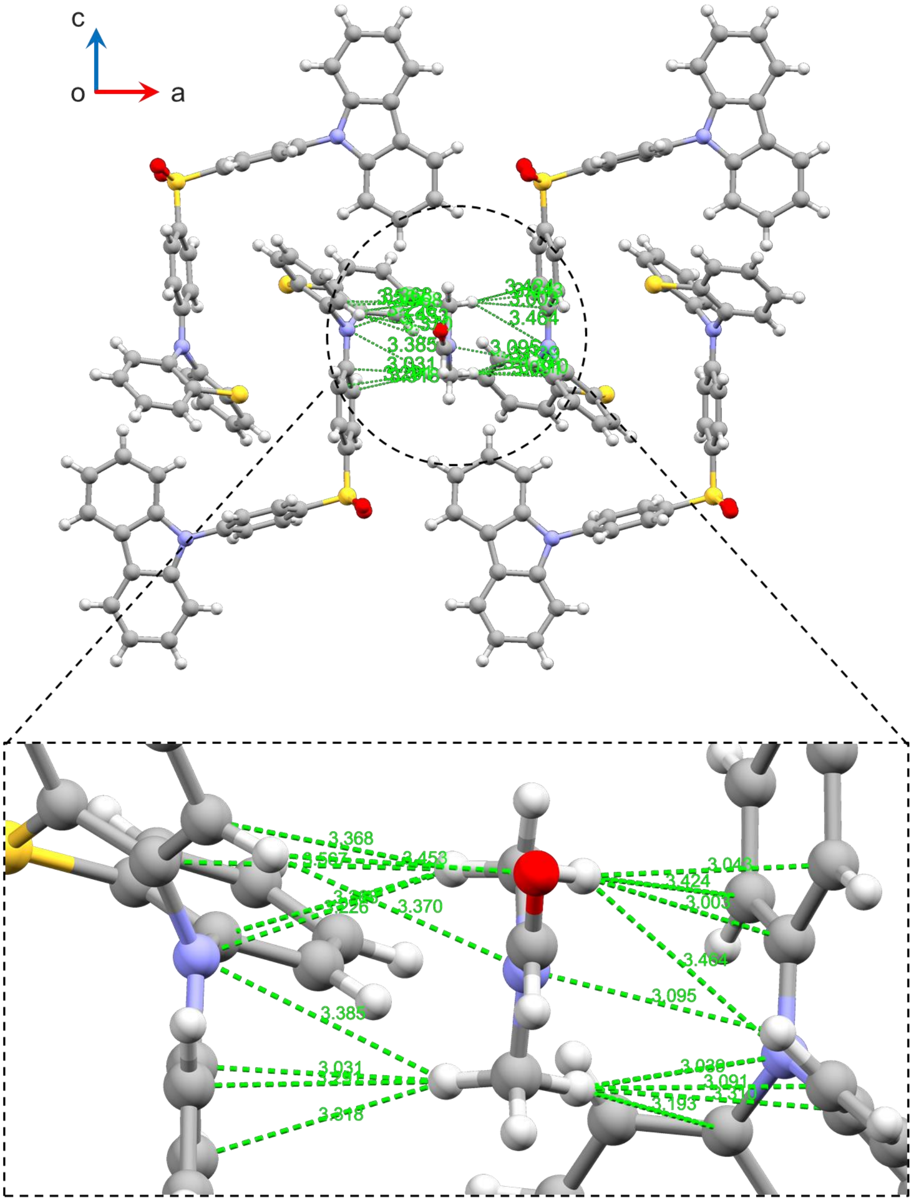

Supplementary Figure 35 | The intermolecular noncovalent interactions between the dish-like molecular architecture and DMF molecule in the single-crystal structure of CP-DMF. Color code: grey, C; red, O; blue, N; yellow, S; white, H.

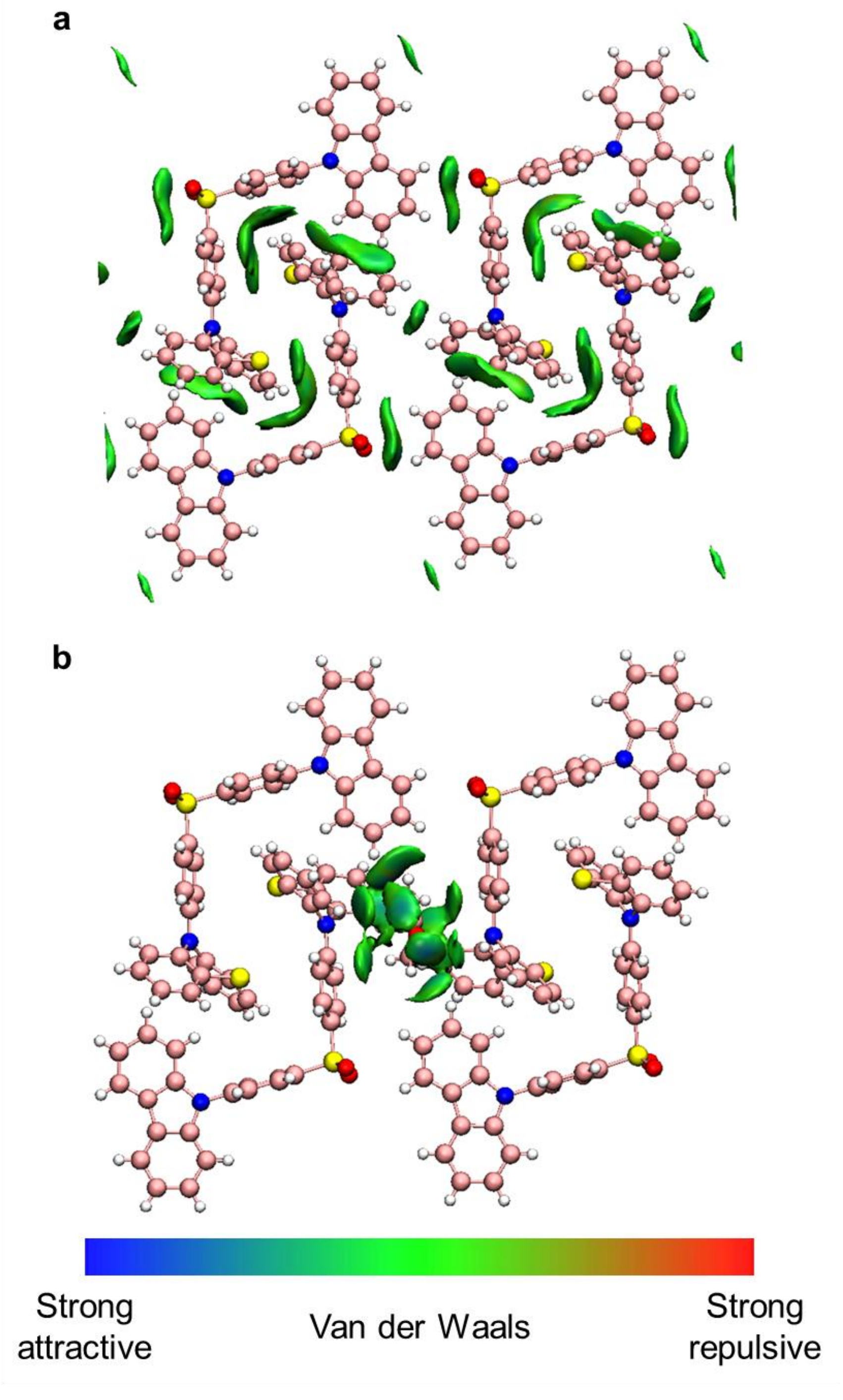

Supplementary Figure 36 | Distribution of intermolecular NCI regions at **a,** molecular architecture locations and **b,** guest locations of the single-crystal structures of CP-DMF, respectively. Color code: pink, C; red, O; blue, N; yellow, S; white, H.

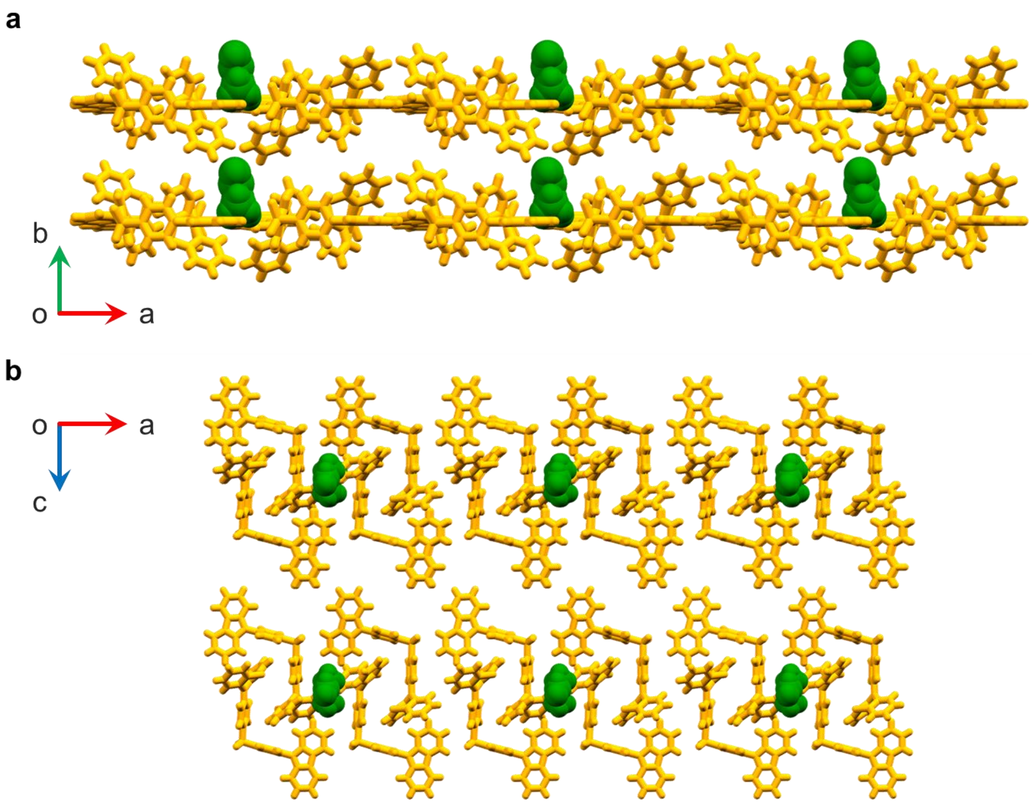

Supplementary Figure 37 | Molecular self-assembly diagrams of CP-DMF viewing along the **a,** *c* axis and **b,** *b* axis. CP and DMF molecules are colored yellow and green, respectively.

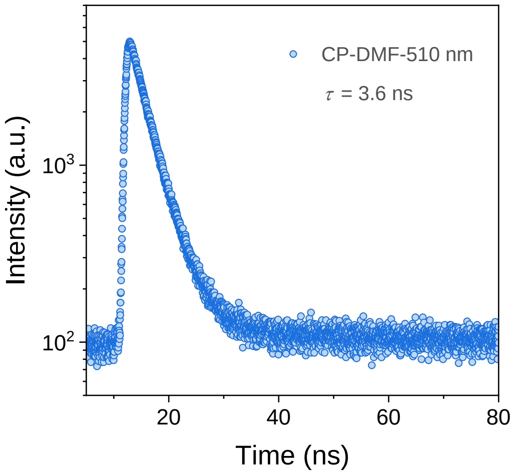

Supplementary Figure 38 | The lifetime decay profile of CP-DMF measured at 510 nm under ambient conditions with a 350 nm nano-LED source.

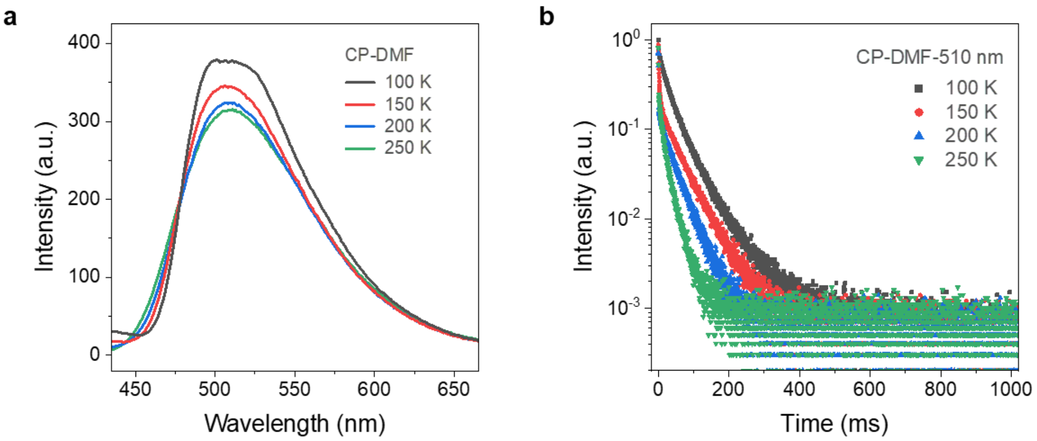

Supplementary Figure 39 | Temperature-dependent **a,** photoluminescence spectra and **b,** lifetime decay profiles of CP-DMF under vacuum conditions with 350 nm excitation wavelength and a 355 nm spectral-LED source. Note: the enhancement of shoulder peaks at 470 nm with the temperature increasing is due to the thermally activated delayed fluorescence (TADF) property of CP-DMF.[5]

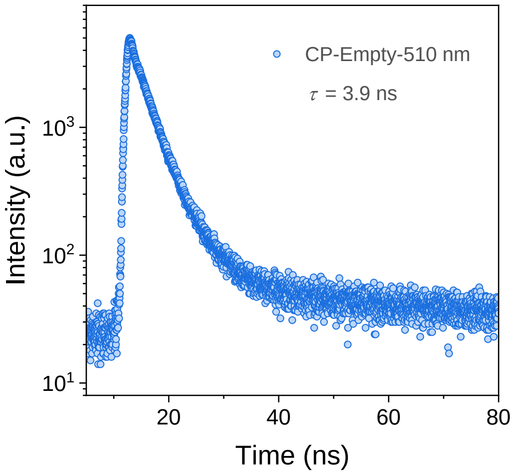

Supplementary Figure 40 | The lifetime decay profile of CP-Empty measured at 510 nm under ambient conditions with a 350 nm nano-LED source.

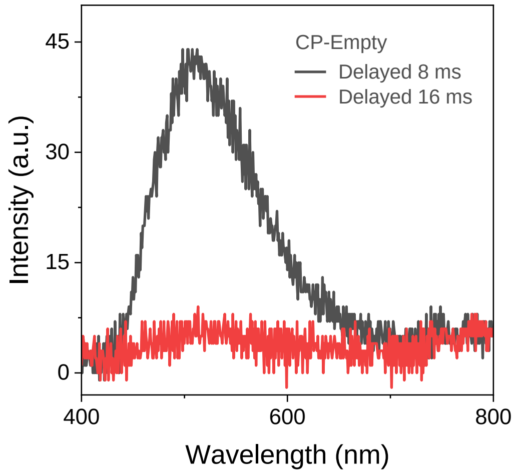

Supplementary Figure 41 | Phosphorescence spectra of CP-Empty crystals with different delay times at room temperature under ambient conditions excited by 365 nm.

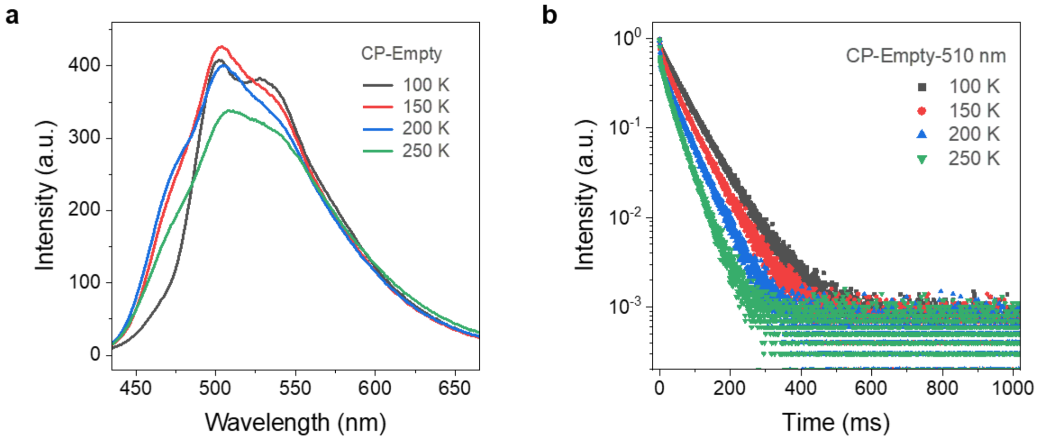

Supplementary Figure 42 | Temperature-dependent **a,** photoluminescence spectra and **b,** lifetime decay profiles of CP-Empty under vacuum conditions with 350 nm excitation wavelength and a 355 nm spectral-LED source. Note: the enhancement of shoulder peaks at 470 nm with the temperature increasing is due to the thermally activated delayed fluorescence (TADF) property of CP-Empty.[5]

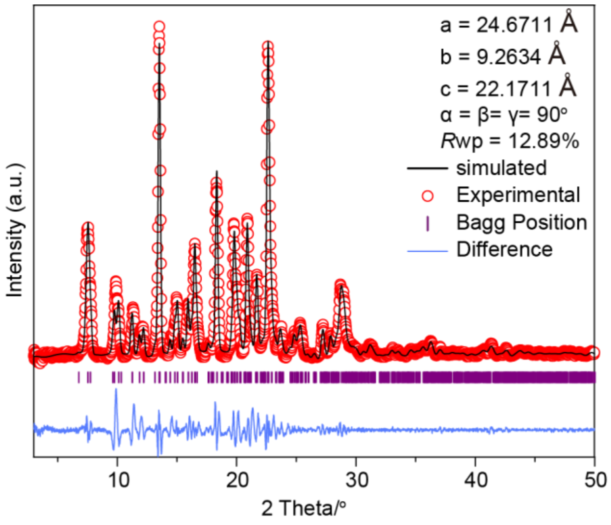

Supplementary Figure 43 | Structural determination of CP-Empty by Pawley refinements based on PXRD patterns. Note: The result resolved by Pawley refinements shows content convergence with Rwp value of 12.89% and the $Pna2_1$ space group with unit-cell parameters of a = 24.6711 Å, b = 9.2634 Å and c = 22.1711 Å, featuring a slight contraction compared to the initial cell parameters of CP-DMF (a = 26.0834 Å, b = 9.76020 Å and c = 23.7023 Å).

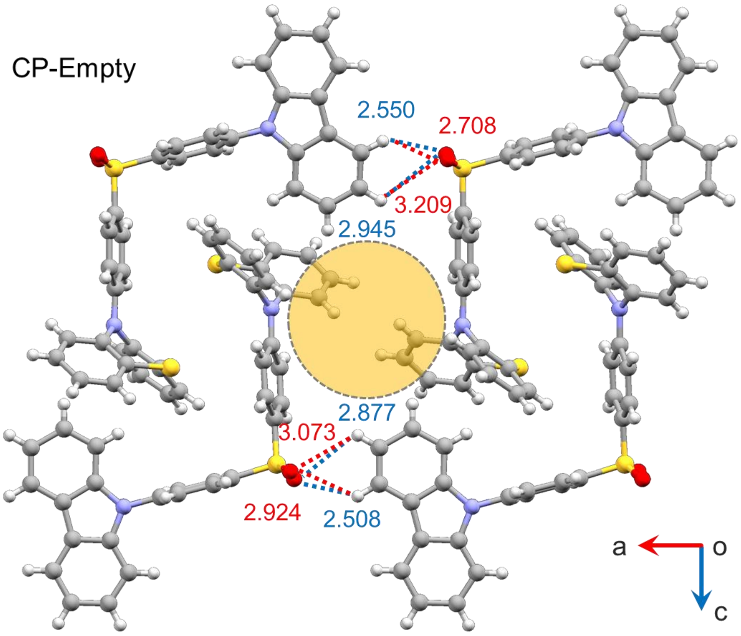

Supplementary Figure 44 | The intermolecular C–H···O=S interactions and cavity of the dish-like molecular architecture in the crystal structure of CP-Empty. Color code: grey, C; red, O; blue, N; yellow, S; white, H.

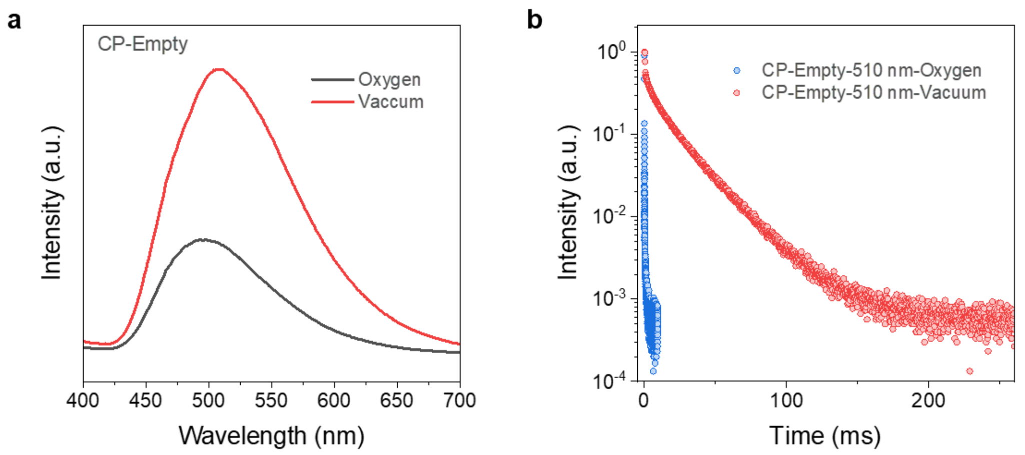

Supplementary Figure 45 | a, photoluminescence spectra and **b,** lifetime decay profiles of CP-Empty under oxygen and vacuum conditions with 350 nm excitation wavelength and a 355 nm spectral-LED source.

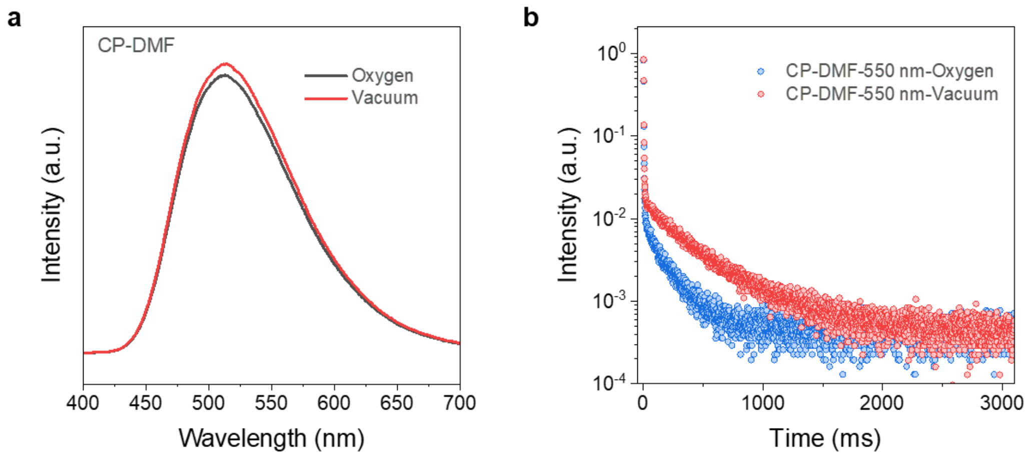

Supplementary Figure 46 | a, photoluminescence spectra and **b,** lifetime decay profiles of CP-DMF under oxygen and vacuum conditions with 350 nm excitation wavelength and a 355 nm spectral-LED source.

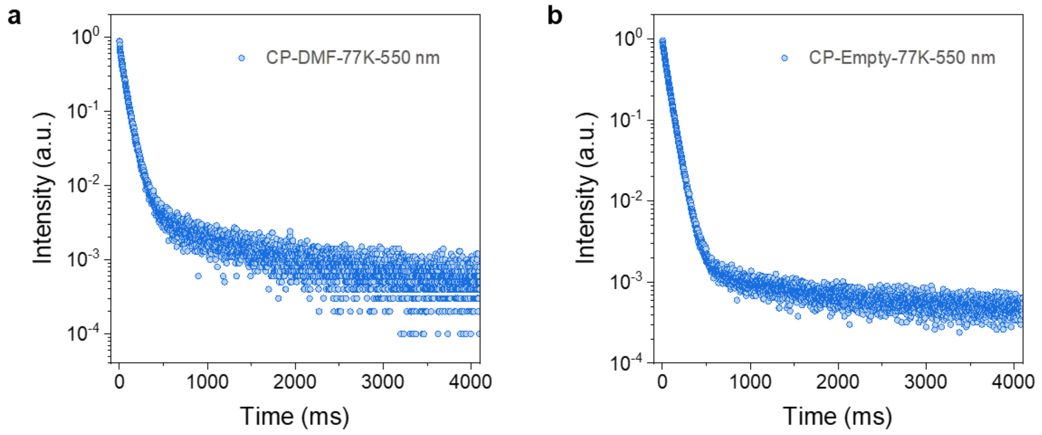

Supplementary Figure 47 | Lifetime decay profiles of **a,** CP-DMF and **b,** CP-Empty at 77 K under vacuum conditions with a 355 nm spectral-LED source.

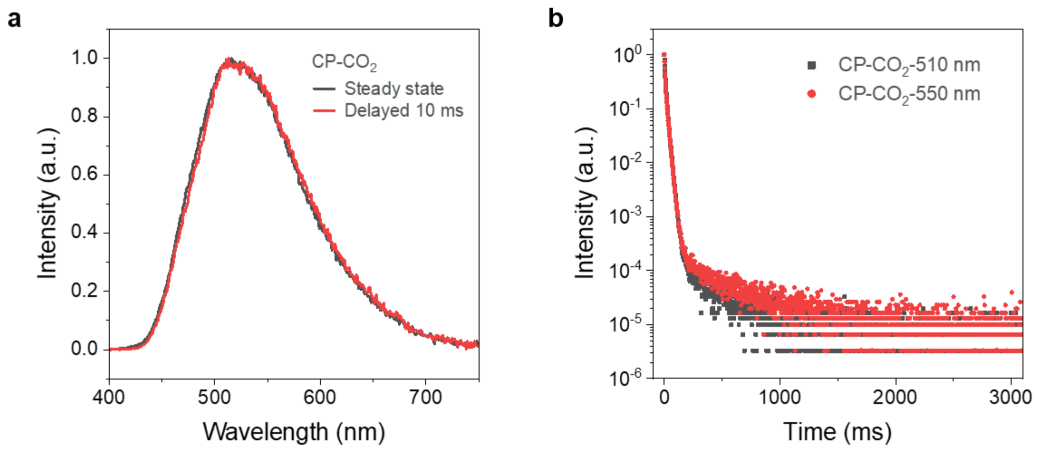

Supplementary Figure 48 | a, The steady photoluminescence spectrum, phosphorescence spectrum and **b,** lifetime decay profiles of CP-CO_2 measured with an excitation wavelength of 365 nm.

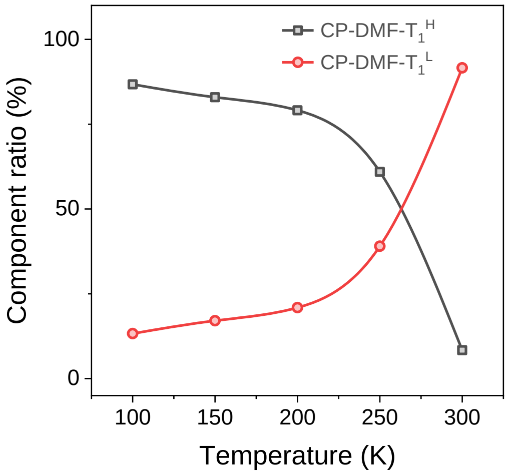

Supplementary Figure 49 | The component ratios of CP-DMF-T_1^H and CP-DMF-T_1^L at different temperature with a 355 nm spectral-LED source.

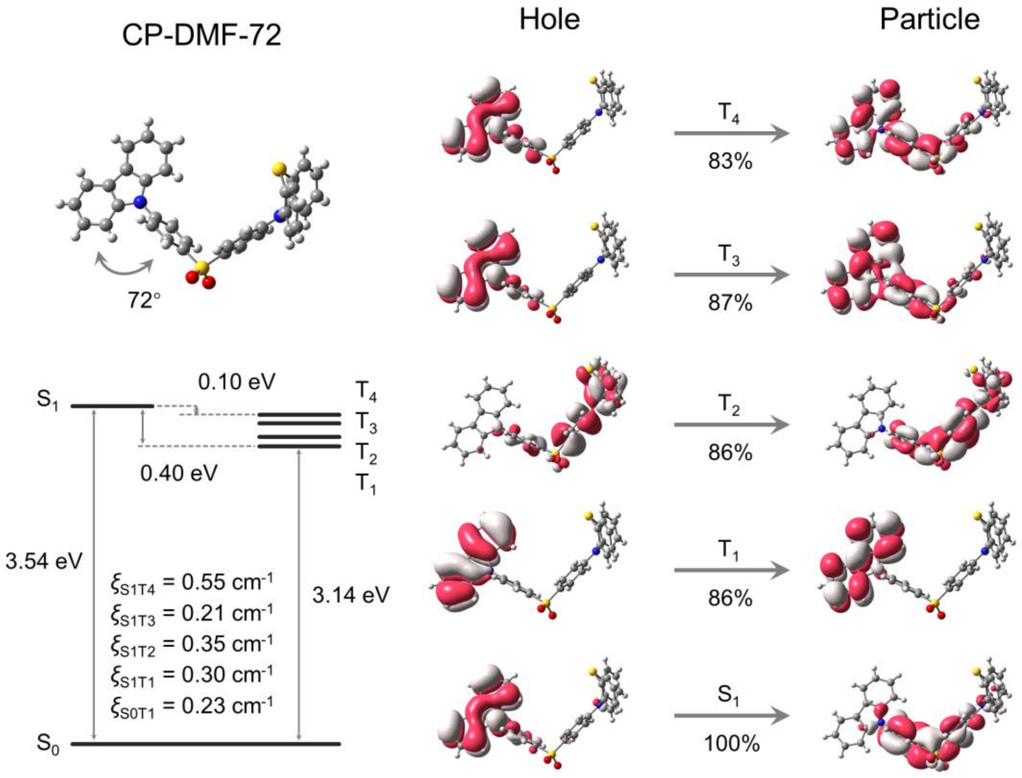

Supplementary Figure 50 | Molecular conformation, calculated energy levels and related spin–orbit coupling (ξ) constants of CP-DMF-72 based on the single-crystal geometry (left). The natural transition orbitals (NTOs) of CP-DMF-72 for S_1, T_1, T_2, T_3 and T_4 with the corresponding proportions in NTOs (right).

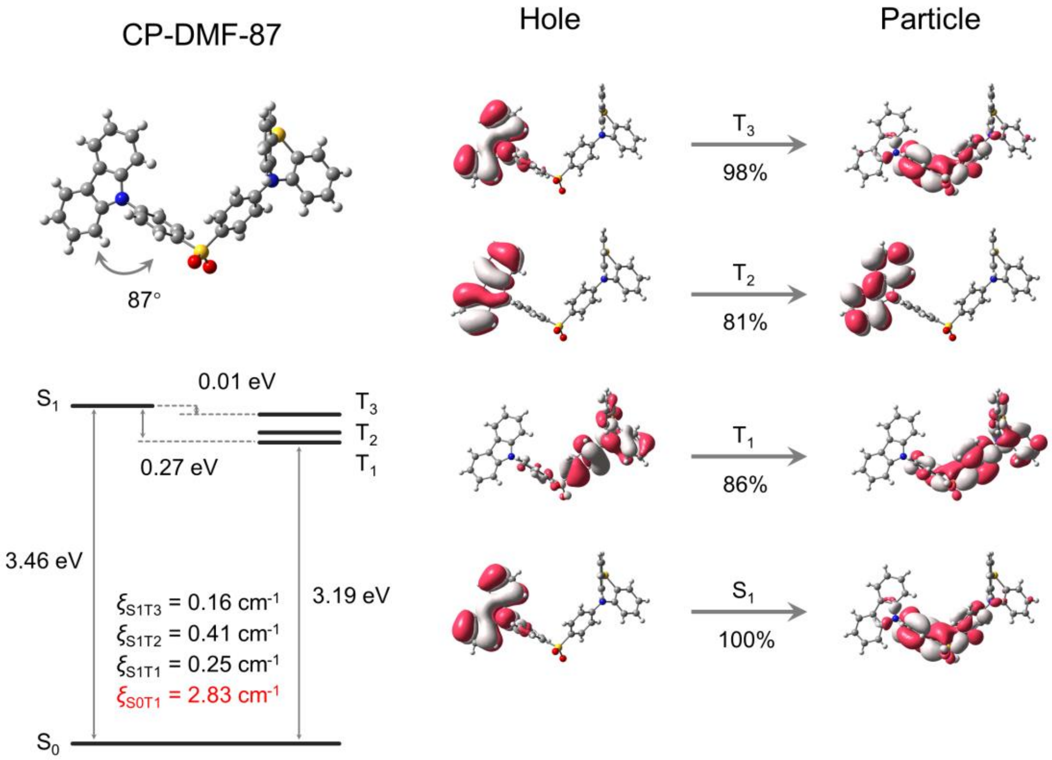

Supplementary Figure 51 | Molecular conformation, calculated energy levels and related spin–orbit coupling (ξ) constants of CP-DMF-87 based on the single-crystal geometry (left). The natural transition orbitals (NTOs) of CP-DMF-87 for S_1, T_1, T_2 and T_3 with the corresponding proportions in NTOs (right).

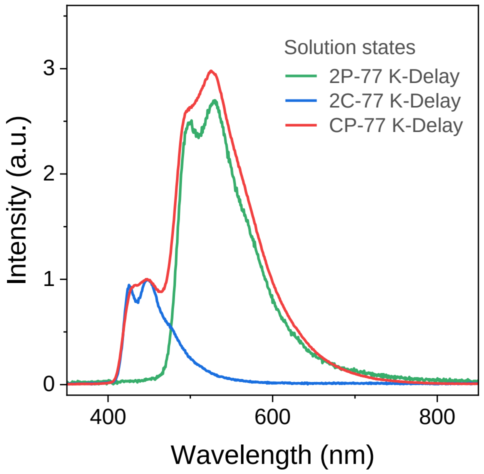

Supplementary Figure 52 | Phosphorescence spectra of 2P, 2C and CP in DCM solutions with 10 μM recorded at 77 K excited by 365 nm. The delay times are 8 ms. Note: The phosphorescence band at 510 nm of CP is identical with that of 2P, further suggesting that the T_1^H with an emission peak at 510 nm in CP-DMF crystals is generated from the phenothiazine-substituted diphenyl sulfone moiety. However, the emission peak at 550 nm in CP-DMF crystals is undetectable in the solution, and as a result, the T_1^L emission peak at 550 nm from the carbazole moiety is regulated and stabilized by the molecular aggregation.[12,13]

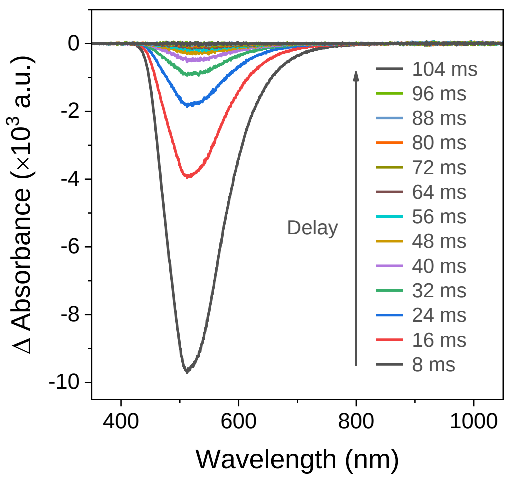

Supplementary Figure 53 | Transient absorption spectra of CP-DMF crystals under ambient conditions excited by 365 nm.

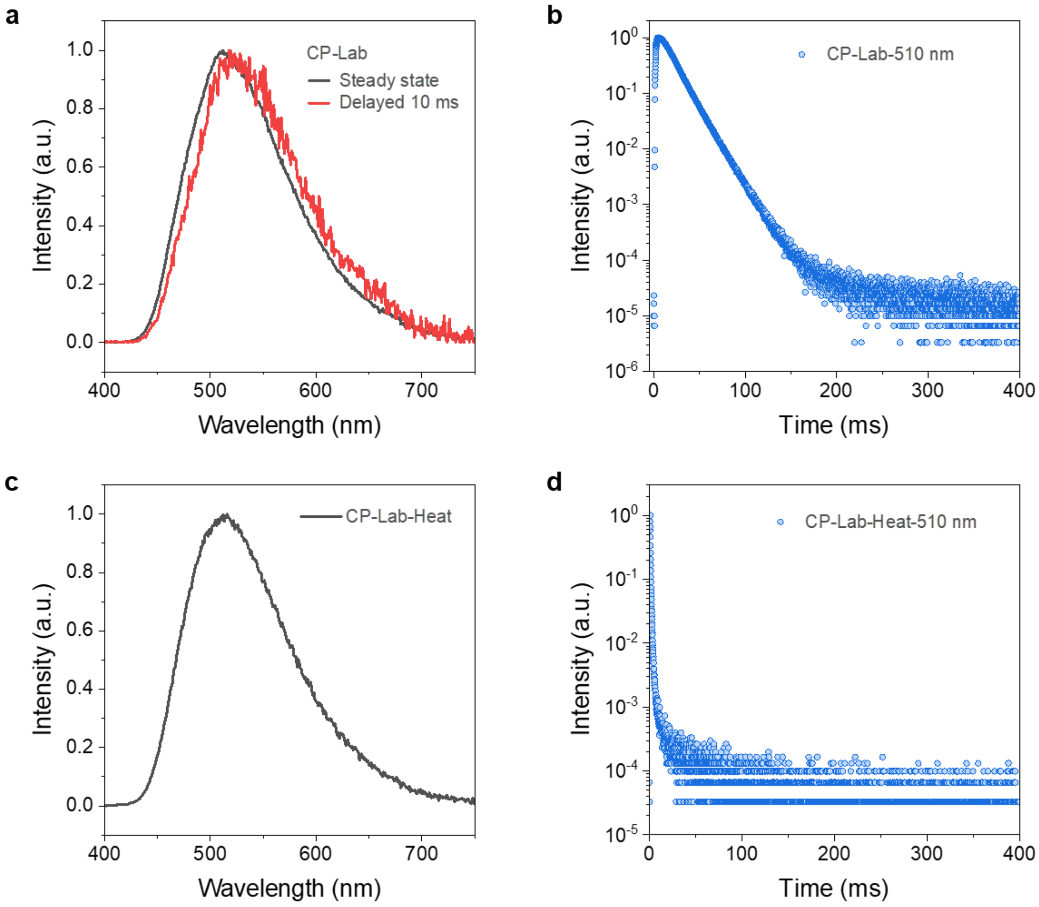

Supplementary Figure 54 | a, The steady photoluminescence spectrum, phosphorescence spectrum and **b,** lifetime decay profile of CP-Lab crystals, and **c,** the steady photoluminescence spectrum and **d,** lifetime decay profile of CP-Lab-Heat crystals under ambient conditions excited by 365 nm. Note: CP-Lab crystals are obtained by the recrystallization in a DCM/EtOH mixed solution.

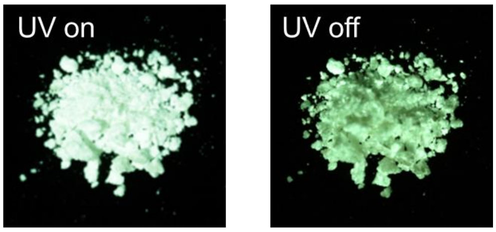

Supplementary Figure 55 | Photographs of CP-Lab crystals taken before and after turning off the UV-light source under ambient conditions.

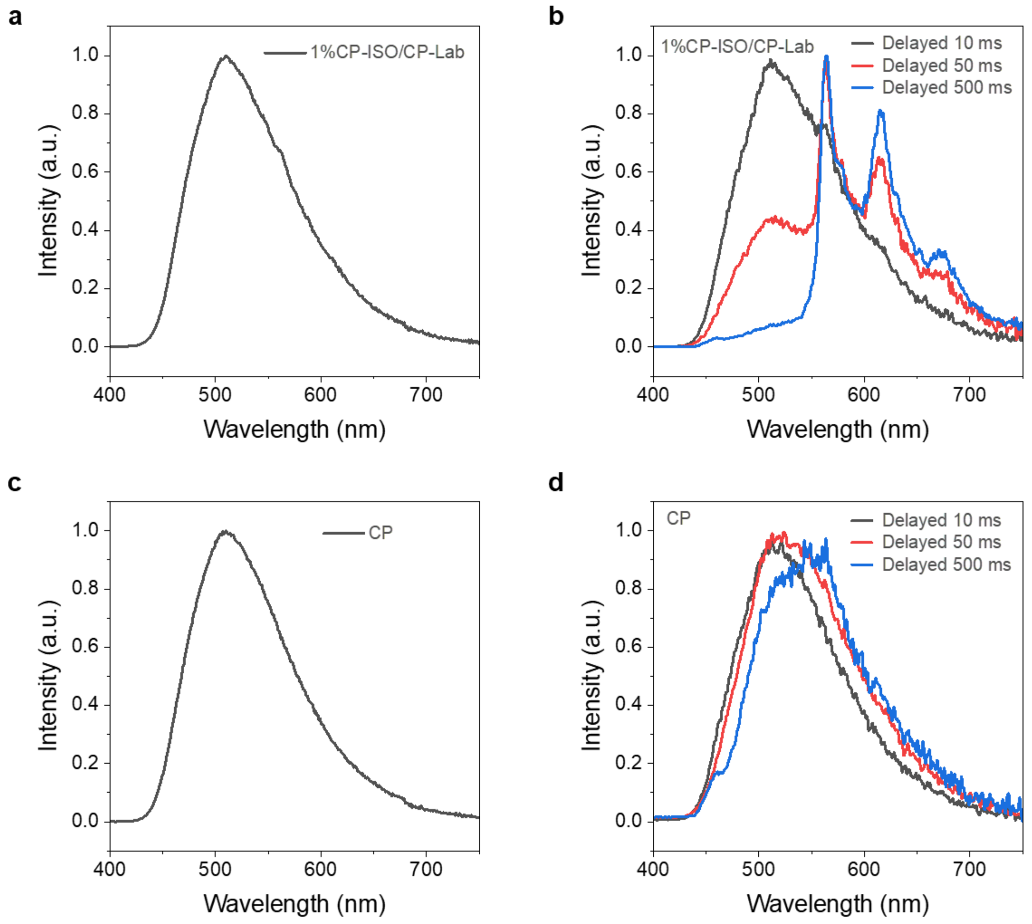

Supplementary Figure 56 | a, The steady photoluminescence spectrum and **b,** phosphorescence spectra at various delay times of 1%CP-ISO/CP-Lab crystals, and **c,** the steady photoluminescence spectrum and **d,** phosphorescence spectra at various delay times of CP crystals under ambient conditions excited by 365 nm. Note: 1%CP-ISO/CP-Lab is prepared by doping 1% CP-ISO into CP-Lab with mass ratio. 1%CP-ISO/CP-Lab crystals are obtained by the recrystallization in a DCM/EtOH mixed solution. CP crystals are obtained by the recrystallization in a DCM/EtOH mixed solution. As shown in Figure S39, the steady PL spectra of 1%CP-ISO/CP-Lab crystals and CP crystals are almost the same. However, their delay PL spectra are quite different. The typical phosphorescence peaks with fine structure of carbazole derivatives synthesized by commercial carbazole can be observed in delay PL spectra of 1%CP-ISO/CP-Lab crystals, of which the maximum emission peak is located at 565 nm. In contrast, only broad phosphorescence peaks are observed in delay PL spectra of CP crystals, which indicates that almost no CP-ISO impurity effect in CP crystals.

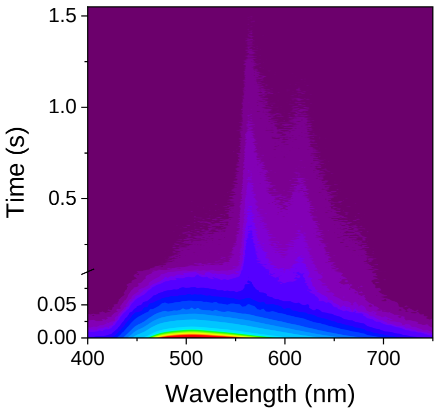

Supplementary Figure 57 | Time-resolved emission spectra of 1%CP-ISO/CP-Lab crystals under ambient conditions with an excitation wavelength of 365 nm.

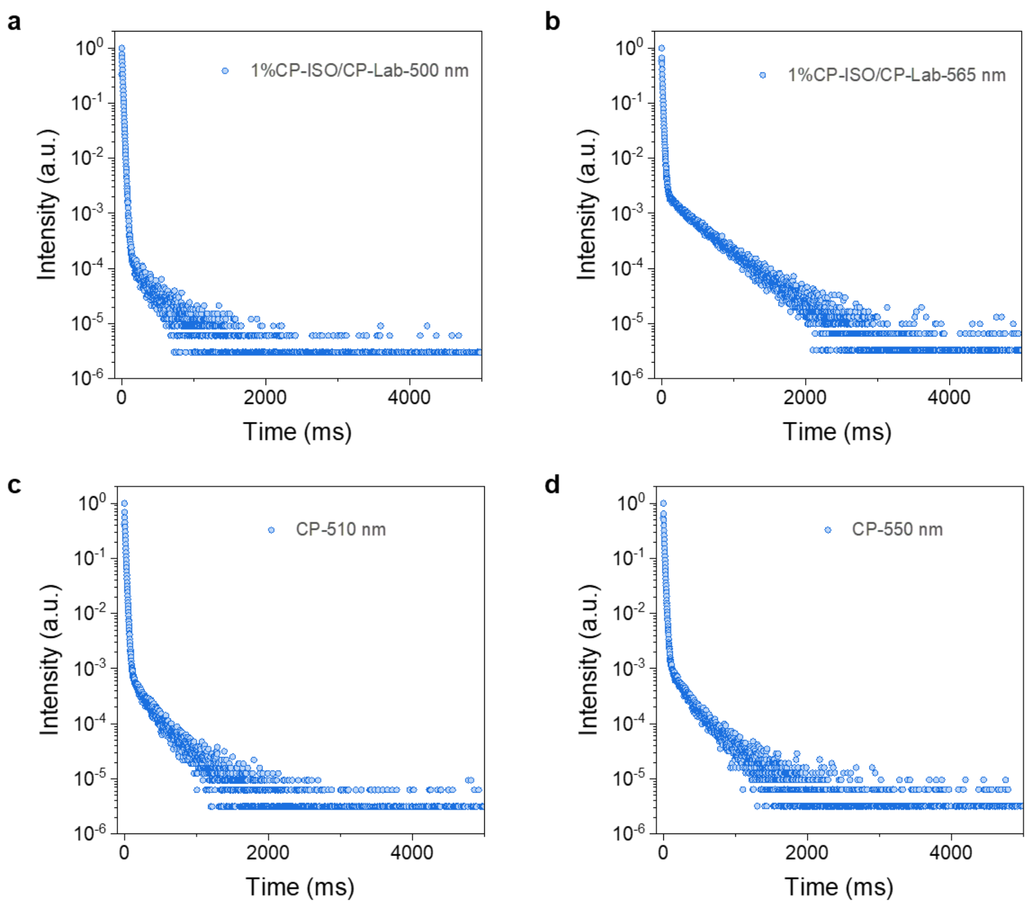

Supplementary Figure 58 | Lifetime decay profiles of 1%CP-ISO/CP-Lab crystals measured at **a,** 500 nm and **b,** 565 nm, respectively. Lifetime decay profiles of CP crystals measured at **c,** 510 nm and **d,** 550 nm, respectively. The excitation wavelength is fixed at 365 nm.

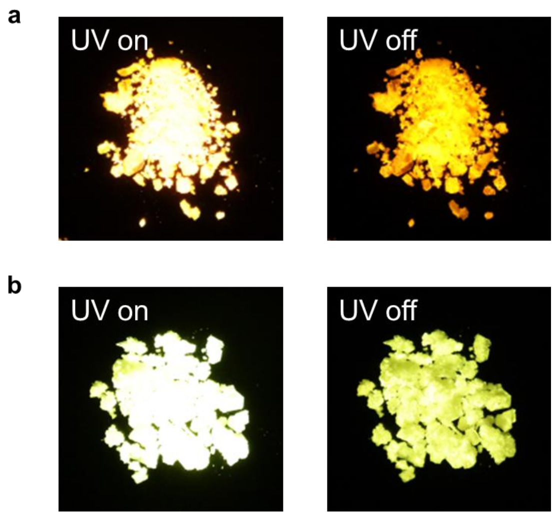

Supplementary Figure 59 | Photographs of **a,** 1%CP-ISO/CP-Lab crystals and **b,** CP crystals taken before and after turning off the UV-light source under ambient conditions, respectively.

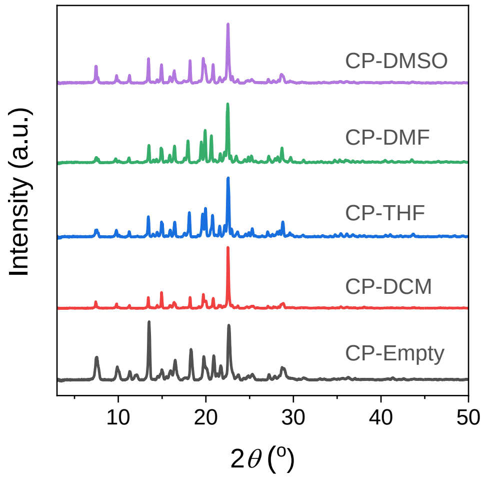

Supplementary Figure 60 | PXRD patterns of CP-DMSO, CP-DMF, CP-THF, CP-DCM and CP-Empty.

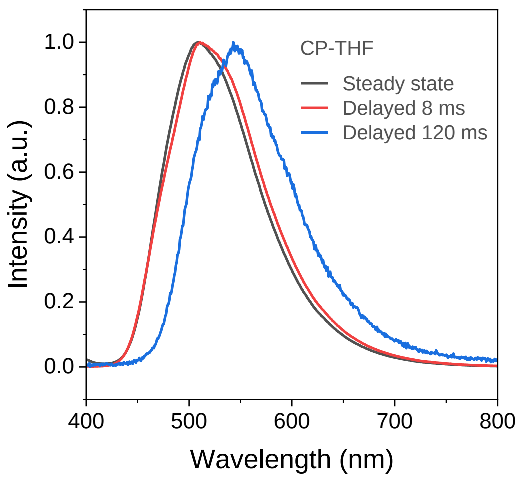

Supplementary Figure 61 | The steady photoluminescence and phosphorescence spectra at delay times of 8 ms and 120 ms of CP-THF under ambient conditions excited by 365 nm.

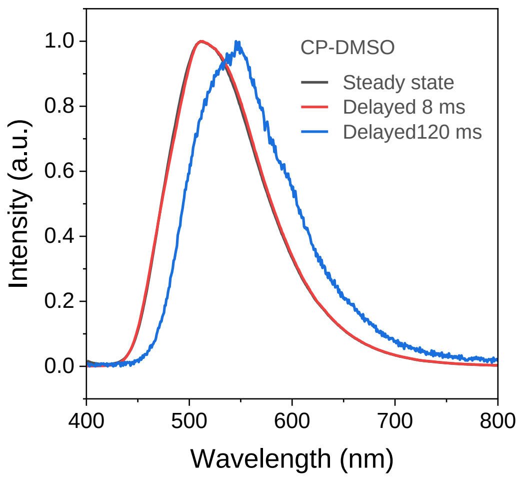

Supplementary Figure 62 | The steady photoluminescence and phosphorescence spectra at delay times of 8 ms and 120 ms of CP-DMSO under ambient conditions excited by 365 nm.

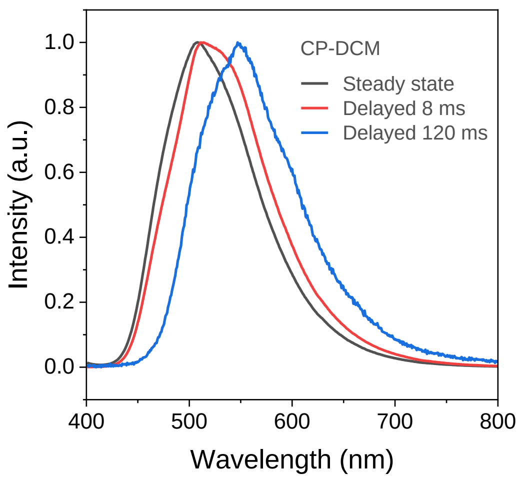

Supplementary Figure 63 | The steady photoluminescence and phosphorescence spectra at delay times of 8 ms and 120 ms of CP-DCM under ambient conditions excited by 365 nm.

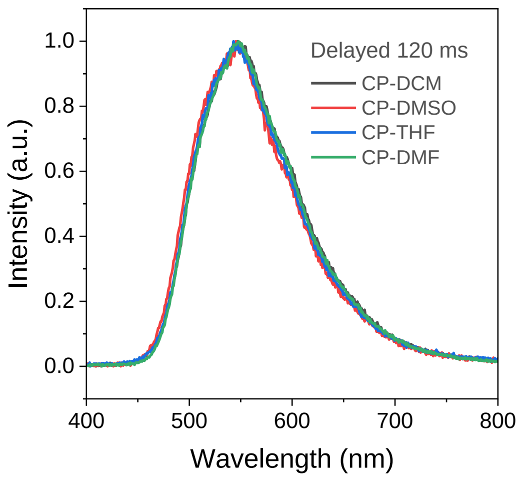

Supplementary Figure 64 | The phosphorescence spectra at a delay time of 120 ms of CP-DCM, CP-DMSO, CP-THF and CP-DMF under ambient conditions excited by 365 nm.

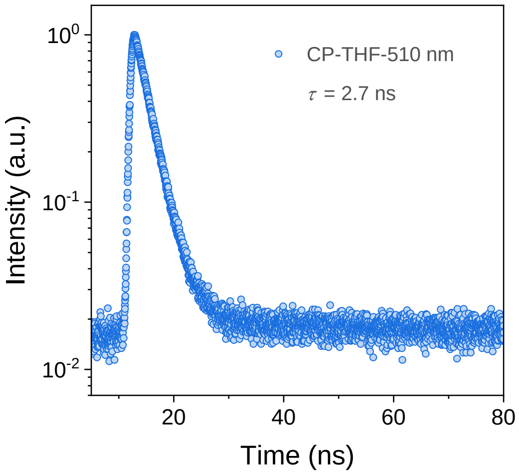

Supplementary Figure 65 | The lifetime decay profile of CP-THF measured at 510 nm under ambient conditions with a 350 nm nano-LED source.

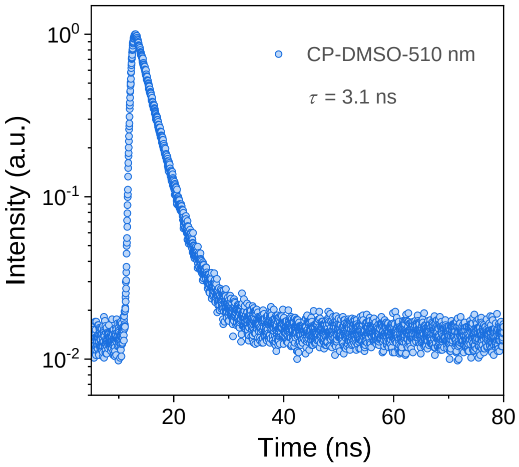

Supplementary Figure 66 | The lifetime decay profile of CP-DMSO measured at 510 nm under ambient conditions with a 350 nm nano-LED source.

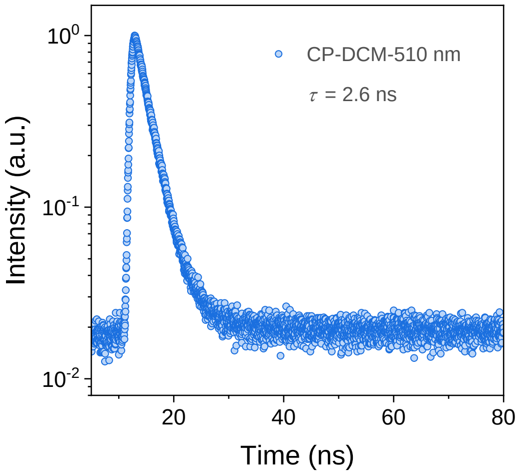

Supplementary Figure 67 | The lifetime decay profile of CP-DCM measured at 510 nm under ambient conditions with a 350 nm nano-LED source.

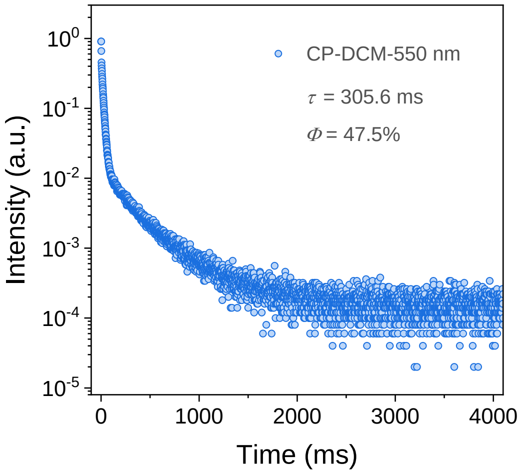

Supplementary Figure 68 | The lifetime decay profile of CP-DCM measured at 510 nm under ambient conditions with a 355 nm spectral-LED source.

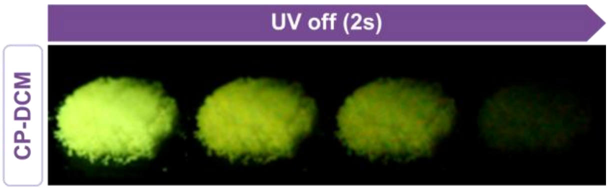

Supplementary Figure 69 | Photographs of RTP phenomenon of CP-DCM crystals under ambient conditions excited at 365 nm.

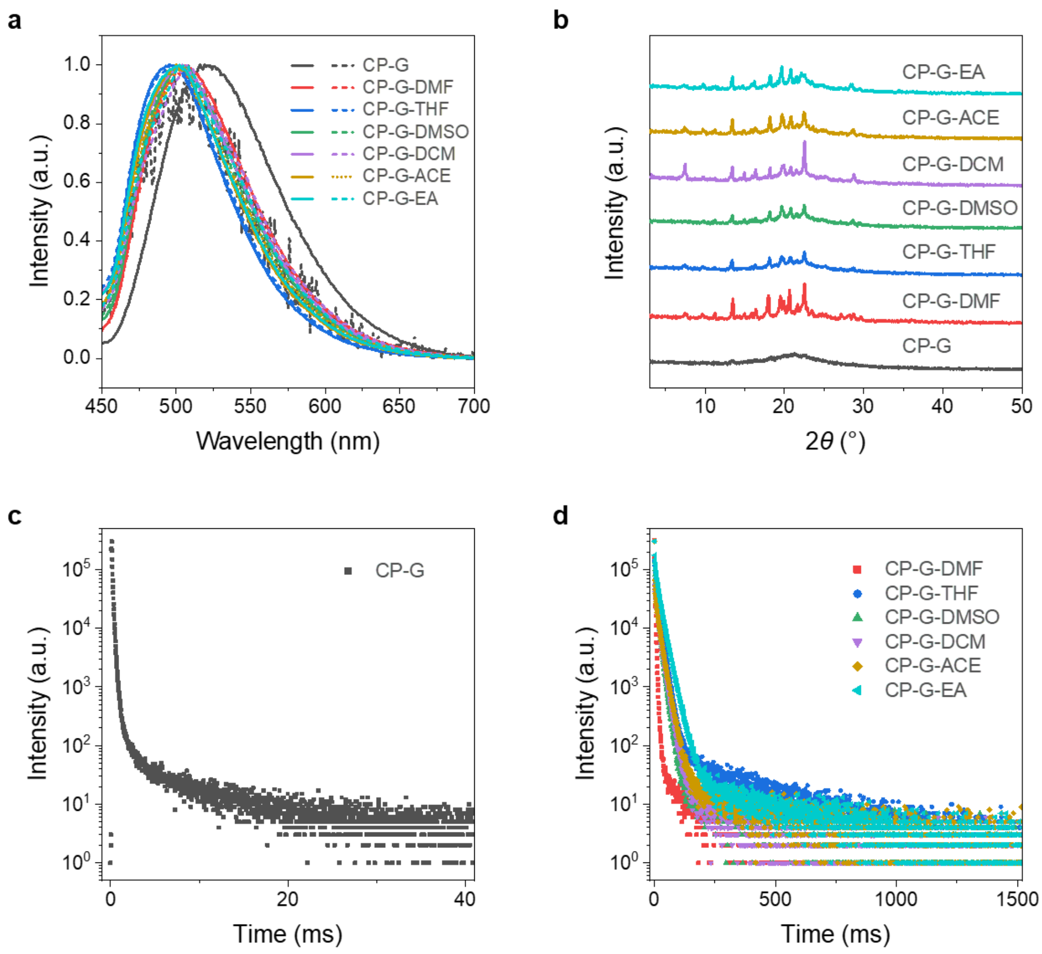

Supplementary Figure 70 | a, steady (solid lines) and delayed (dash lines) photoluminescence spectra and **b,** PXRD of CP-G, CP-G-DMF, CP-G-THF, CP-G-DMSO, CP-G-DCM, CP-G-ACE and CP-G-EA, respectively. ACE: acetone. EA: ethyl acetate. For the delayed spectra, except the delay time of CP-G is 5 ms, delay times of other samples are 10 ms. The lifetime decay profiles of c, CP-G, d, CP-G-DMF, CP-G-THF, CP-G-DMSO, CP-G-DCM, CP-G-ACE and CP-G-EA, respectively. The excitation wavelength is fixed at 375 nm. Note: Through grinding samples, amorphous state of CP can be obtained (named CP-G), which can be verified by PXRD. Compared to CP crystals, the steady luminescence of CP-G shows a red-shift emission with a 520 nm peak and a much shorter lifetime of phosphorescence. After fumed with various solvents, respectively, all CP-G samples completed the transformation from amorphous states to crystalline states, showing vivid URTP and long lifetimes again.

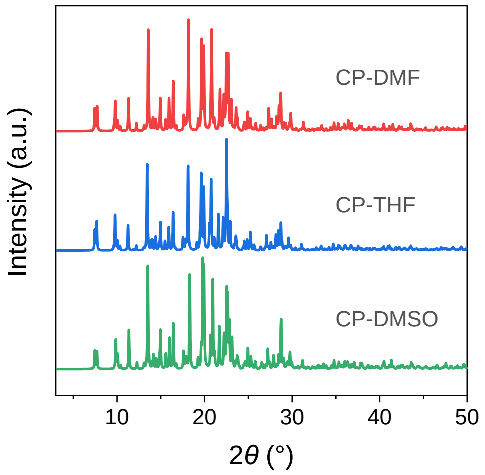

Supplementary Figure 71 | Simulated PXRD patterns of CP-DMF, CP-THF, and CP-DMSO according to their single crystals.

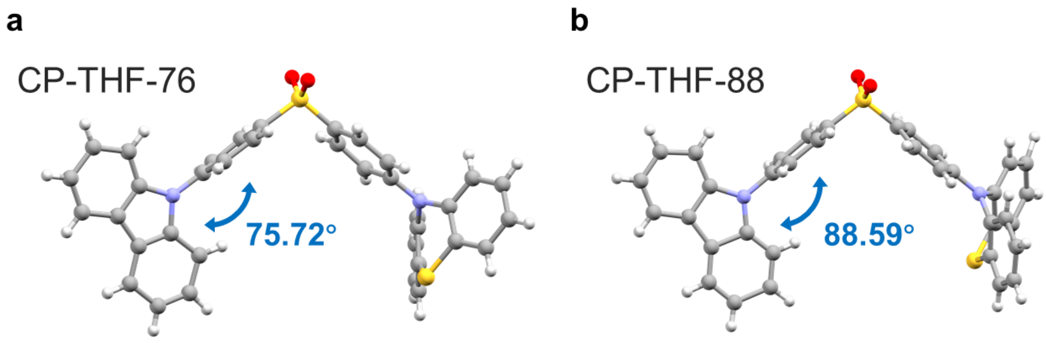

Supplementary Figure 72 | Two crystallographically independent CP molecules in the single-crystal structure of CP-THF, in which the dihedral angles between the plane of the carbazole moiety and the plane of the adjacent phenyl ring are **a,** 75.72° and **b,** 88.59°, respectively. Color code: grey, C; red, O; blue, N; yellow, S; white, H.

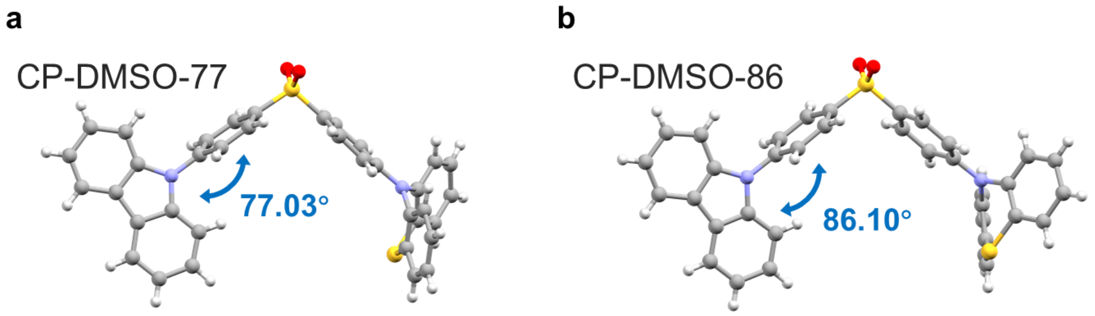

Supplementary Figure 73 | Two crystallographically independent CP molecules in the single-crystal structure of CP-DMSO, in which the dihedral angles between the plane of the carbazole moiety and the plane of the adjacent phenyl ring are **a,** 77.03° and **b,** 86.10°, respectively. Color code: grey, C; red, O; blue, N; yellow, S; white, H.

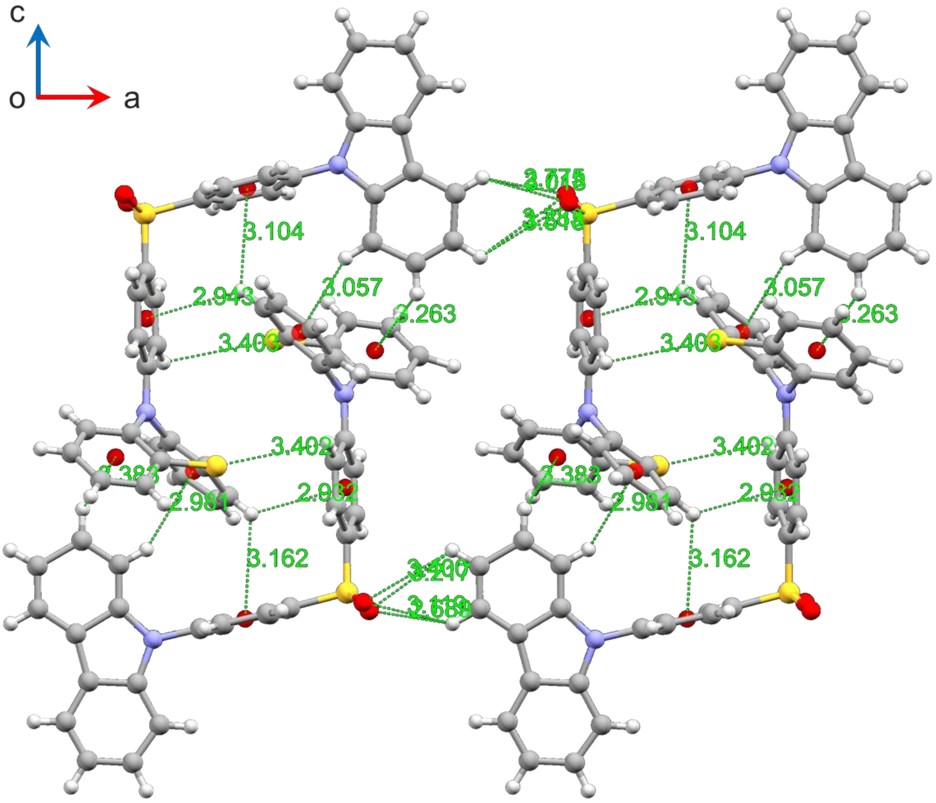

Supplementary Figure 74 | The intermolecular noncovalent interactions of the dish-like molecular architecture in the single-crystal structure of CP-THF. Color code: grey, C; red, O; blue, N; yellow, S; white, H.

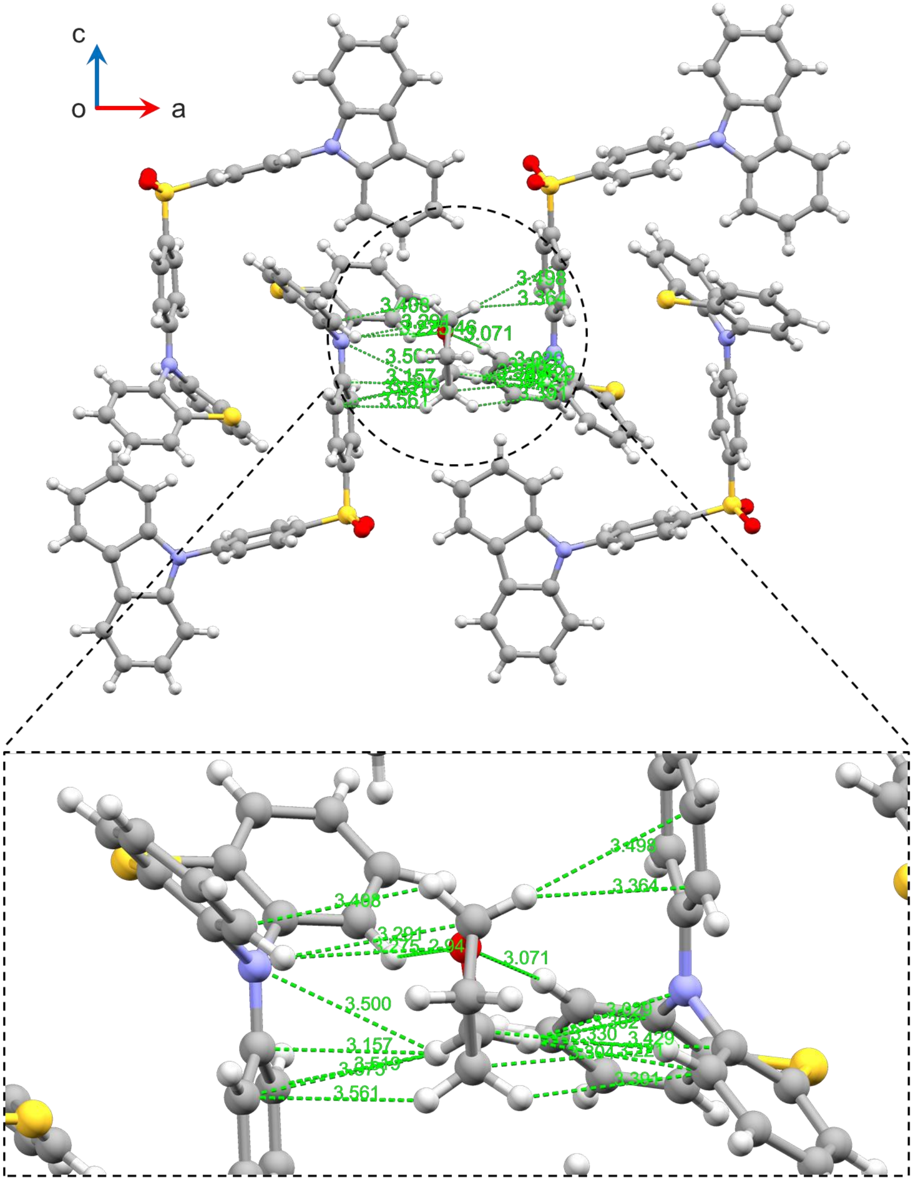

Supplementary Figure 75 | The intermolecular noncovalent interactions between the dish-like molecular architecture and THF molecule in the single-crystal structure of CP-THF. Color code: grey, C; red, O; blue, N; yellow, S; white, H.

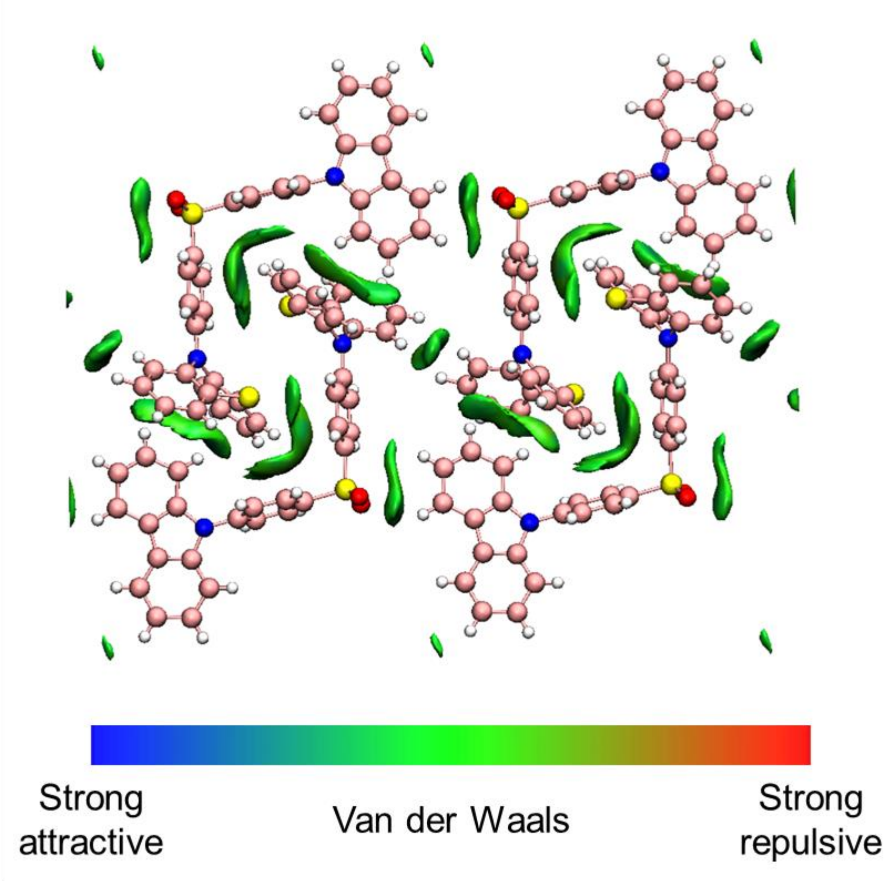

Supplementary Figure 76 | Distribution of intermolecular NCI regions at molecular architecture locations of the single-crystal structures of CP-THF. Color code: pink, C; red, O; blue, N; yellow, S; white, H.

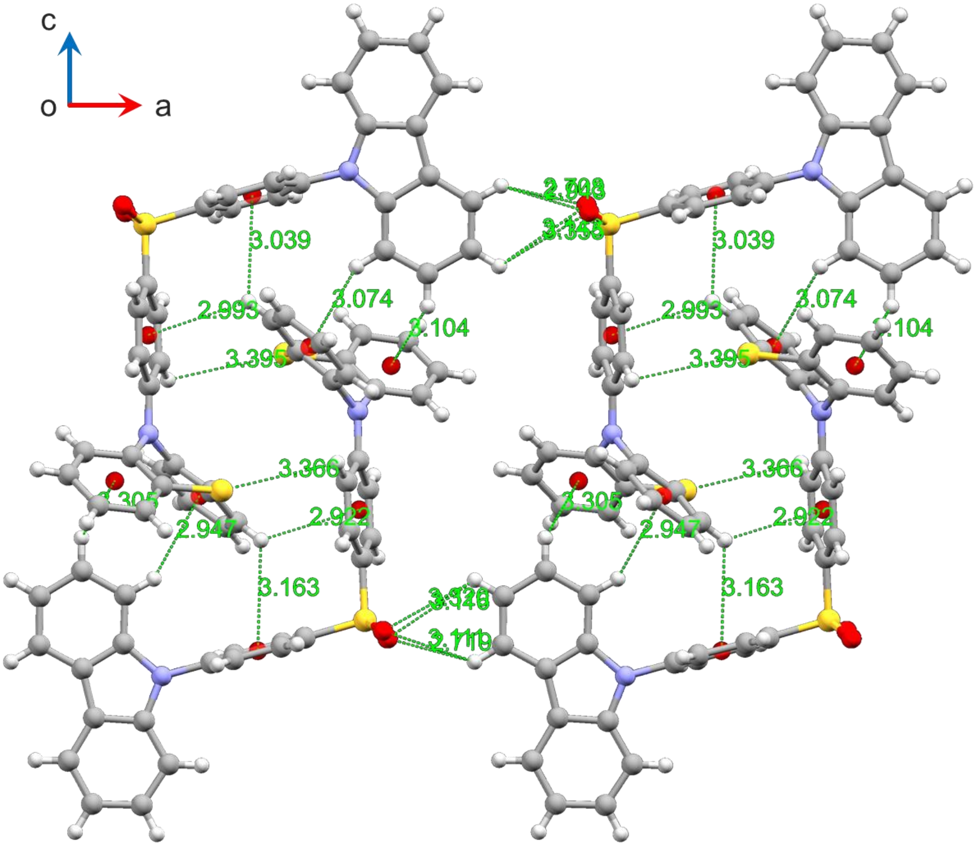

Supplementary Figure 77 | The intermolecular noncovalent interactions of the dish-like molecular architecture in the single-crystal structure of CP-DMSO. Color code: grey, C; red, O; blue, N; yellow, S; white, H.

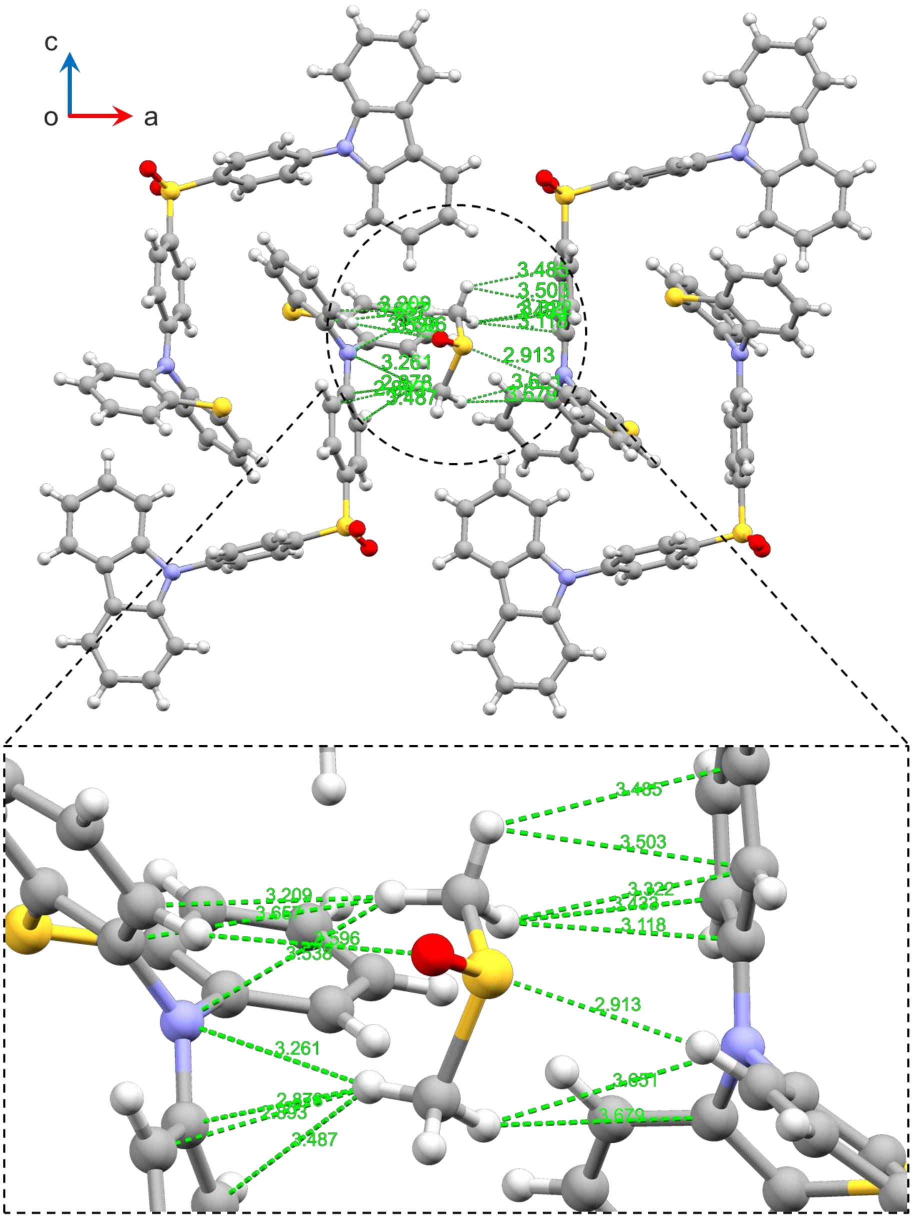

Supplementary Figure 78 | The intermolecular noncovalent interactions between the dish-like molecular architecture and THF molecule in the single-crystal structure of CP-DMSO. Color code: grey, C; red, O; blue, N; yellow, S; white, H.

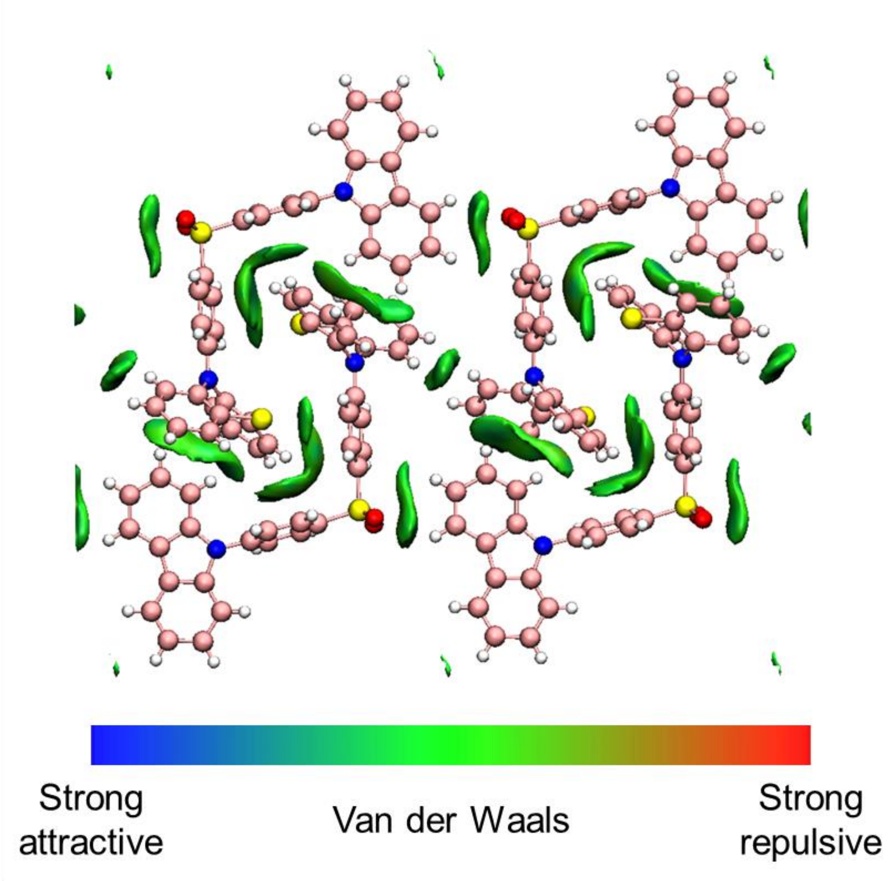

Supplementary Figure 79 | Distribution of intermolecular NCI regions at molecular architecture locations of the single-crystal structures of CP-DMSO. Color code: pink, C; red, O; blue, N; yellow, S; white, H.

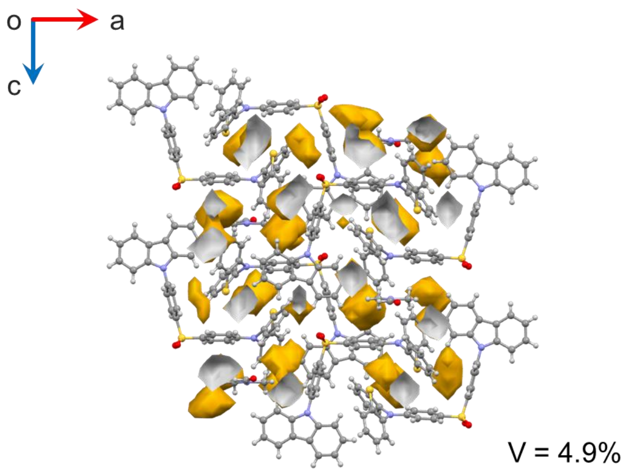

Supplementary Figure 80 | Single-crystal structure of CP-DMF along the *b* axis, with the solvent-accessible void space visualized by gray/yellow (inner/outer) curved planes generated with a probe of 0.7 Å. Color code: grey, C; red, O; blue, N; yellow, S; white, H.

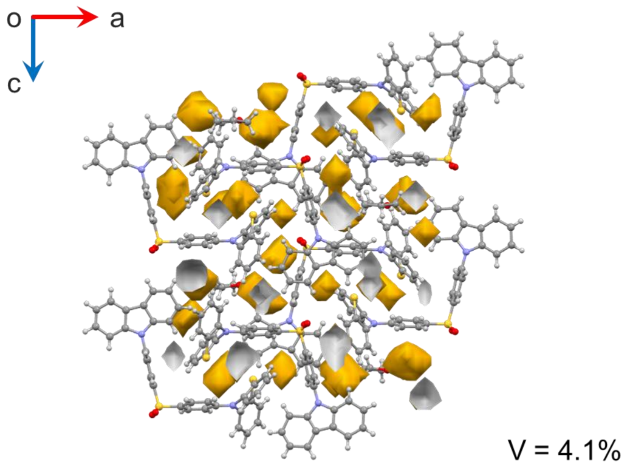

Supplementary Figure 81 | Single-crystal structure of CP-THF along the *b* axis, with the solvent-accessible void space visualized by gray/yellow (inner/outer) curved planes generated with a probe of 0.7 Å. Color code: grey, C; red, O; blue, N; yellow, S; white, H.

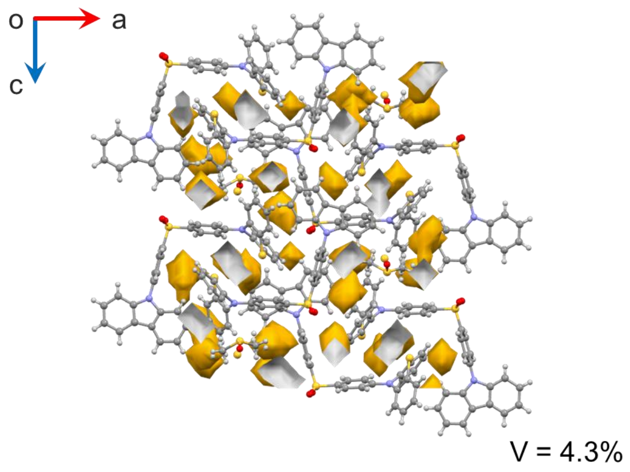

Supplementary Figure 82 | Single-crystal structure of CP-DMSO along the *b* axis, with the solvent-accessible void space visualized by gray/yellow (inner/outer) curved planes generated with a probe of 0.7 Å. Color code: grey, C; red, O; blue, N; yellow, S; white, H.

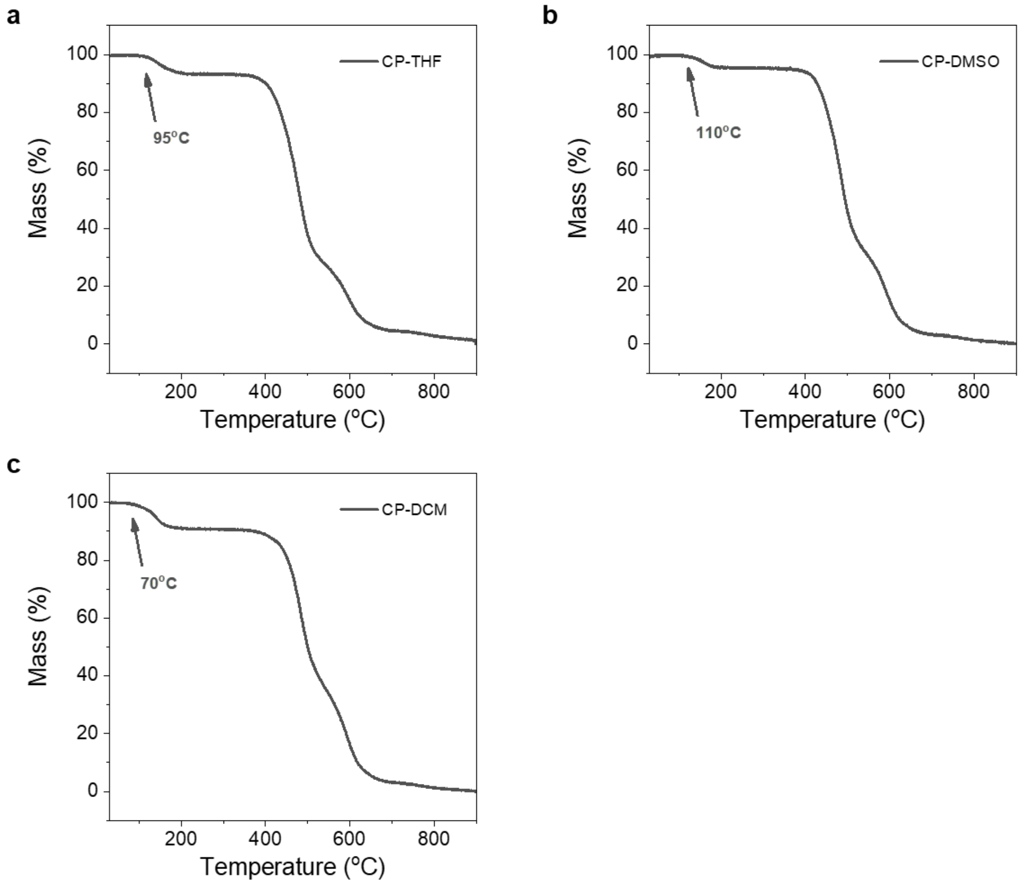

Supplementary Figure 83 | TGA curves of **a,** CP-THF, **b,** CP-DMSO and **c,** CP-DCM crystals, respectively.

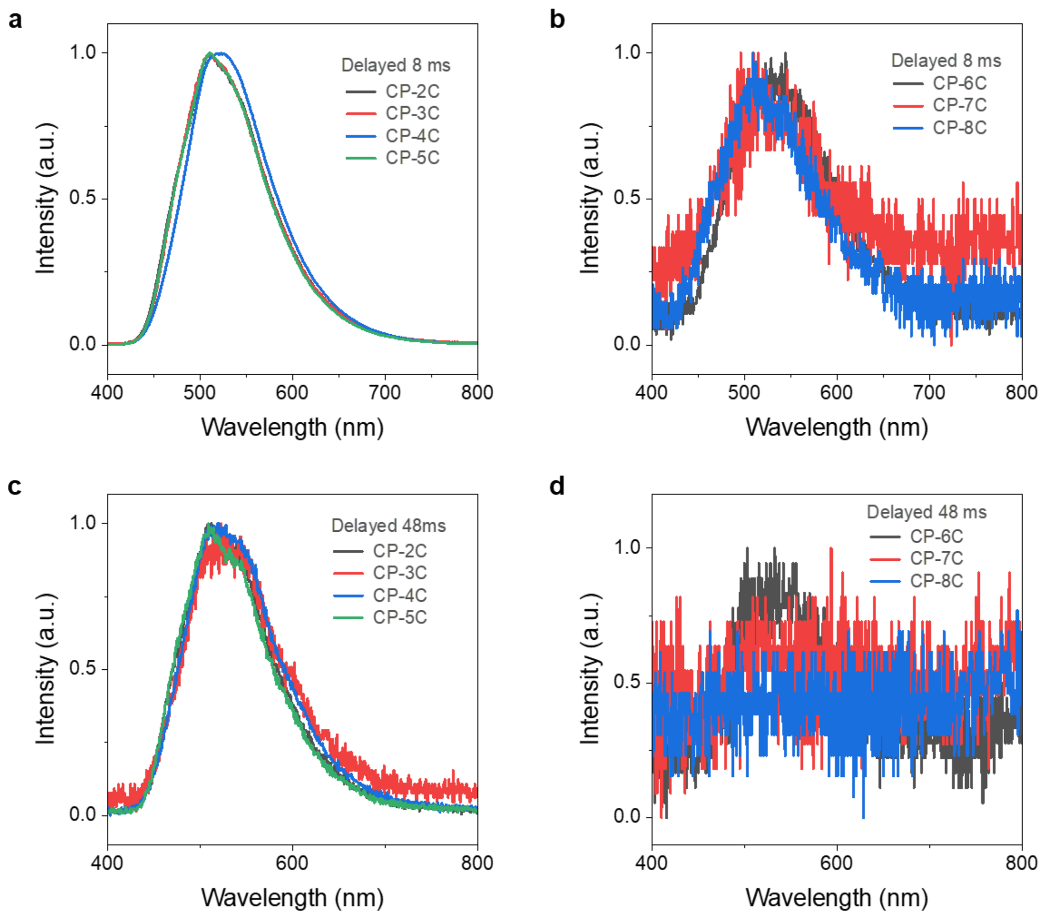

Supplementary Figure 84 | Phosphorescence spectra with a delay time of **a,** 8 ms and **c,** 48 ms in CP-2C, CP-3C, CP-4C and CP-5C, respectively. Phosphorescence spectra with a delay time of **b,** 8 ms and **d,** 48 ms in CP-6C, CP-7C and CP-8C, respectively. The excitation wavelength is fixed at 365 nm. Note: owing to the external heavy atom effect, lifetimes of CP-6C, CP-7C and CP-8C are slightly longer than that of CP-Empty.

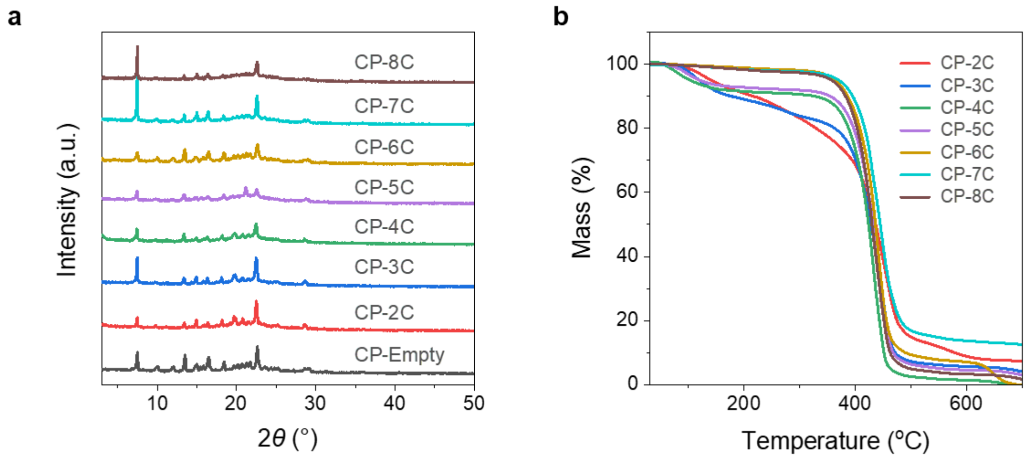

Supplementary Figure 85 | a, PXRD patterns of CP-XC (X = 2 to 8) and CP-Empty. **b,** TGA curves of CP-XC (X = 2 to 8). Note: The same PXRD patterns indicate the dish-like molecular architectures of CP are still maintained after fuming with 2C to 8C, respectively. The mass losses of CP-2C to CP-5C in low temperature area as shown in TGA curves reveal the accommodation of 2C to 5C in cavity of CP-Empty, while 6C to 8C can not be accommodated due to the larger size.

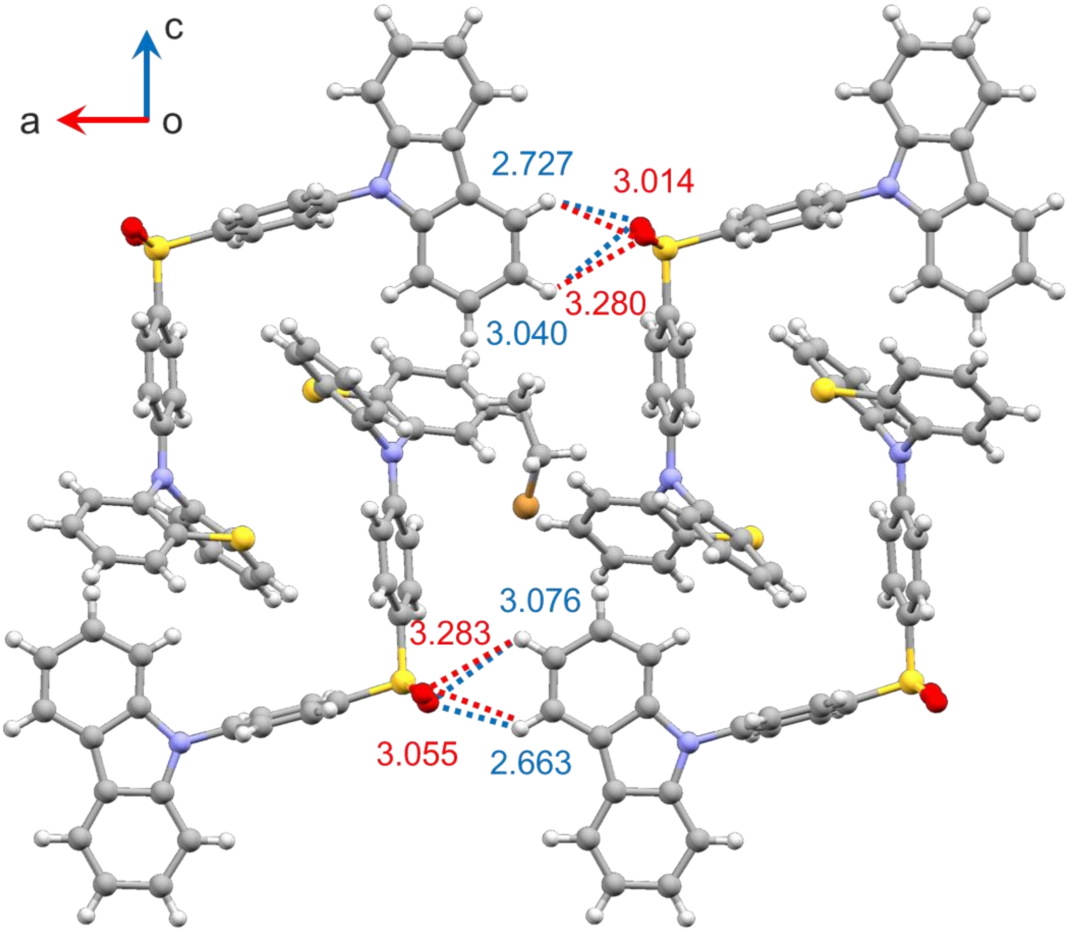

Supplementary Figure 86 | The intermolecular C–H···O=S interactions in the crystal structure of CP-2C. Color code: grey, C; red, O; blue, N; yellow, S; white, H; brown, Br.

Supplementary Tables

Supplementary Table 1 | Crystallographic data for the single crystal of CP-DMF, CP-THF and CP-DMSO.

**Single crystal**	**CP-DMF**	**CP-THF**	**CP-DMSO**
Formula	$C_{37.5}H_{27.5}N_{2.5}O_{2.5}S_2$	$C_{38}H_{28}N_2O_{2.5}S_2$	$C_{37}H_{27}N_2O_{2.5}S_{2.5}$
Formula weight	617.24	616.74	619.75
Temperature (K)	150.00(10)	226.05(10)	223.00(10)
Crystal system	orthorhombic	orthorhombic	orthorhombic
Space group	$Pna2_1$	$Pna2_1$	$Pna2_1$
a (Å)	26.0834(4)	26.3302(11)	26.1849(3)
b (Å)	9.76020(10)	9.7874(4)	9.68440(10)
c (Å)	23.7023(3)	23.6727(11)	23.6682(3)
α (°)	90	90	90
β (°)	90	90	90
γ (°)	90	90	90
V (Å^3)	6034.11(13)	6100.6(5)	6001.90(12)
Z	8	8	8
F (000)	2576.0	2576.0	2584.0
D_c (g·cm^{-3})	1.359	1.343	1.372
Radiation (CuKα)	λ = 1.54184	λ = 1.54184	λ = 1.54184
Reflections collected	16034	15392	32005
Unique reflns	9177	8109	10584
Parameters	804	808	805
R_{int}	0.0249	0.0254	0.0461
μ/ mm^{-1}	1.925	1.899	2.249
R_1 $[I \geq 2\sigma(I)]^a$	0.0558	0.0575	0.0856
wR_2 $[I \geq 2\sigma(I)]^b$	0.1548	0.1592	0.2126
R_1 (all data)	0.0578	0.0613	0.0924
wR_2 (all data)	0.1573	0.1646	0.2235
GOF	1.037	1.050	1.072

^{a} $R_1=\Sigma||F_o|-|F_c||/\Sigma|F_o|$.

^{b} $wR_2=[\Sigma w(F_o^2-F_c^2)^2/\Sigma w(F_o^2)^2]^{1/2}$.

Supplementary Table 2 | Data of intermolecular noncovalent interactions of the dish-like molecular architecture in the single-crystal structure of CP-DMF.

Type	Distance (Å)
C–H···O	2.653
	2.695
	2.863
	3.050
	3.092
	3.124
	3.253
	3.398
C–H···S	3.319 × 2
	3.492 × 2
C–H···π	2.925 × 2
	2.959 × 2
	3.054 × 2
	3.067 × 2
	3.086 × 2
	3.125 × 2
	3.217 × 2
	3.239 × 2

Supplementary Table 3 | Data of intermolecular noncovalent interactions between the dish-like molecular architecture and DMF molecule in the single-crystal structure of CP-DMF.

Type	Distance (Å)
O–H···C	3.003
	3.043
	3.424
O–H···N	3.464
C–H···O	3.453
C–H···N	3.039
	3.095
	3.226
	3.370
	3.385
C–H···C	3.031
	3.091
	3.193
	3.291
	3.310
	3.318
	3.368
	3.368
	3.567

**Supplementary Table 4 | ** Data of temperature-dependent lifetime decay profiles of CP-DMF and CP-Empty.

**Temperature (K)**	**Lifetime (ms)**	
	**CP-DMF**	**CP-Empty**
100	55.1	59.2
150	53.7	49.4
200	39.2	45.6
250	23.6	37.1

**Supplementary Table 5 | ** Photophysical parameters of CP-Empty, CP-DMF, CP-THF, CP-DMSO and CP-DCM.

Sample	CP-Empty	CP-DMF	CP-THF	CP-DMSO	CP-DCM
Φ_{total} (%) [a]	11.2	72.2	85.1	75.5	51.6
τ_F (ns)	3.9	3.6	2.7	3.1	2.6
Φ_F (%)	6.4	11.5	6.3	12.8	4.1
τ_P (ms) / A(%) [b]	0.02 / 22.18	2.2 / 15.95	1.7 / 7.43	2.2 / 17.03	2.1 / 8.00
	0.7 / 34.78	12.3 / 22.15	16.7 / 34.95	14.2 / 52.38	18.7 / 64.09
	3.8 / 43.04	348.0 / 61.90	378.9 / 57.62	483.1 / 30.59	305.6 / 27.91
Φ_P (%) [c]	4.8	60.7	78.8	62.7	47.5
$k_{nr,P}$ (s^{-1}) [a]	251.8	31.9	12.7	26.2	28.0
k_{ISC} (s^{-1}) [a]	1.2×10^7	1.7×10^8	2.9×10^8	2.0×10^8	1.8×10^8

[a] $\Phi_{total} = \Phi_F + \Phi_P$; $k_{nr,p} = (1-\Phi_P) / \tau_P$; $k_{ISC} = \Phi_P / \tau_F$.

[b] Determined from the fitting function of $I(t) = A_1e^{-t/\tau1} + A_2e^{-t/\tau2} + A_3e^{-t/\tau3}$ according to phosphorescence decay profiles.

[c] Φ_P were calculated by the phosphorescence component of relevant lifetimes.[11]

**Supplementary Table 6 | ** Data of lifetime decay profiles of CP-DMF and CP-Empty in various conditions.

**Condition**	**Lifetime (ms)**	
	**CP-DMF**	**CP-Empty**
Oxygen	2.6 (20.92%)	0.03 (38.13%)
	138.7 (79.08)	0.5 (61.87%)
Vacuum	2.9 (6.77%)	5.2 (16.63%)
	302.9 (93.23)	21.8 (83.37%)
77 K	62.6 (88.49%)	64.7 (96.46%)
	620.0 (11.51%)	594.9 (3.54%)

Supplementary Table 7 | Data of lifetime decay profiles of CP-CO_2.

**CP-CO_2**	**510 nm**	**550 nm**
Lifetime (ms)	5.7 (29.39%)<br>18.2 (70.12%)<br>252.8 (0.50%)	5.7 (30.49%)<br>18.3 (68.68%)<br>384.3 (0.83%)

Supplementary Table 8 | Data of temperature-dependent lifetime decay profiles and component ratios of CP-DMF-T_1^H and CP-DMF-T_1^L, respectively.

**Temperature (K)**	**Lifetime (ms)**	
	**CP-DMF-T_1^H**	**CP-DMF-T_1^L**
100	49.8 (86.73%)	843.9 (13.27%)
150	49.4 (82.93%)	779.6 (17.07%)
200	37.6 (79.08%)	762.5 (20.92%)
250	20.8 (60.96%)	649.3 (39.04%)
300	4.0 (8.39%)	404.0 (91.61%)

Supplementary Table 9 | Data of calculated energy levels and related spin–orbit coupling (ξ) constants of CP-DMF-72 and CP-DMF-87, respectively.

**CP-DMF-72**		**CP-DMF-87**	
Energy level (eV)	ξ (cm^{-1})	Energy level (eV)	ξ (cm^{-1})
$S_0 \rightarrow S_1$ (3.54)	$S_0 \leftrightarrow T_1$ (0.23)	$S_0 \rightarrow S_1$ (3.46)	$S_0 \leftrightarrow T_1$ (2.83)
$S_0 \rightarrow T_1$ (3.14)	$S_1 \leftrightarrow T_1$ (0.30)	$S_0 \rightarrow T_1$ (3.19)	$S_1 \leftrightarrow T_1$ (0.25)
$S_0 \rightarrow T_2$ (3.18)	$S_1 \leftrightarrow T_2$ (0.35)	$S_0 \rightarrow T_2$ (3.28)	$S_1 \leftrightarrow T_2$ (0.41)
$S_0 \rightarrow T_3$ (3.38)	$S_1 \leftrightarrow T_3$ (0.21)	$S_0 \rightarrow T_3$ (3.45)	$S_1 \leftrightarrow T_3$ (0.16)
$S_0 \rightarrow T_4$ (3.44)	$S_1 \leftrightarrow T_4$ (0.55)		

Supplementary Table 10 | Data of intermolecular noncovalent interactions of the dish-like molecular architecture in the single-crystal structure of CP-THF.

Type	Distance (Å)
C–H···O	2.689
	2.775
	3.018
	3.119
	3.217
	3.283
	3.400
	3.518
C–H···S	3.402 × 2
	3.403 × 2
C–H···π	2.932 × 2
	2.943 × 2
	2.981 × 2
	3.057 × 2
	3.104 × 2
	3.162 × 2
	3.263 × 2
	3.383 × 2

Supplementary Table 11 | Data of intermolecular noncovalent interactions between the dish-like molecular architecture and THF molecule in the single-crystal structure of CP-THF.

Type	Distance (Å)
C–H···O	2.946
	3.071
	3.275
C–H···N	3.029
	3.500
C–H···C	3.157
	3.221
	3.291
	3.302
	3.304
	3.330
	3.364
	3.375
	3.391
	3.408
	3.429
	3.498
	3.519
	3.561

Supplementary Table 12 | Data of intermolecular noncovalent interactions of the dish-like molecular architecture in the single-crystal structure of CP-DMSO.

Type	Distance (Å)
C–H···O	2.708
	2.719
	2.943
	3.111
	3.116
	3.143
	3.320
	3.355
C–H···S	3.366 × 2
	3.395 × 2
C–H···π	2.922 × 2
	2.947 × 2
	2.993 × 2
	3.039 × 2
	3.074 × 2
	3.104 × 2
	3.163 × 2
	3.305 × 2

Supplementary Table 13 | Data of intermolecular noncovalent interactions between the dish-like molecular architecture and DMSO molecule in the single-crystal structure of CP-DMSO.

Type	Distance (Å)
C–H···S	2.913
C–H···O	3.596
C–H···N	3.261
	3.538
C–H···C	2.878
	2.893
	3.118
	3.209
	3.322
	3.432
	3.485
	3.487
	3.503
	3.651
	3.657
	3.679

Supplementary References

1. Frisch, M. J. et al. Gaussian, Inc., Wallingford CT (2016).
2. Lu, T. & Chen, F. Multiwfn: a multifunctional wavefunction analyzer. *J. Comput. Chem.* **33**, 580–592 (2012).
3. Zhang, Y.; et al. BDF: a relativistic electronic structure program package. *J. Chem. Phys.* **152**, 064113 (2020).
4. Gao, X. et al. Evaluation of spin-orbit couplings with linear-response time-dependent density functional methods. *J. Chem. Theory Comput.* **13**, 515–524 (2017).
5. Li, W. et al. Selective expression of chromophores in a single molecule: soft organic crystals exhibiting full-colour tunability and dynamic triplet-exciton behaviours. *Angew. Chem. Int. Ed.* **59**, 3739–3745 (2020).
6. Li, W. et al. Activating versatile mechanoluminescence in organic host–guest crystals by controlling exciton transfer. *Angew. Chem. Int. Ed.* **59**, 22645–22651 (2020).
7. Li, J.-A. et al. Colour-tunable dual-mode afterglows and helical-array-induced mechanoluminescence from AIE enantiomers: effects of molecular arrangement on formation and decay of excited states. *Chem. Eng. J.* **418**, 129167 (2021).
8. Chen, J. et al. Achievement of persistent and efficient organic room-temperature phosphorescence with temperature-response by adjusting the proportion of excited-state configurations in coupled molecules. *J. Mater. Chem. C* **7**, 8250–8254 (2019).
9. Yang, Z. et al. Intermolecular electronic coupling of organic units for efficient persistent room-temperature phosphorescence. *Angew. Chem. Int. Ed.* **55**, 2181–2185 (2016).
10. Mao, Z. et al. The methylation effect in prolonging the pure organic room temperature phosphorescence lifetime. *Chem. Sci.* **10**, 179–184 (2019).
11. Jin, J. et al. Thermally activated triplet exciton release for highly efficient tri-mode organic afterglow. *Nat. Commun.* **11**, 842 (2020).
12. An, Z. et al. Stabilizing triplet excited states for ultralong organic phosphorescence. *Nat. Mater.* **14**, 685–690 (2015).
13. Chen, J. et al. Synergistic generation and accumulation of triplet excitons for efficient ultralong organic phosphorescence. *Angew. Chem. Int. Ed.* **61**, e202200343 (2022).
